# Normative theory of visual receptive fields

**DOI:** 10.1016/j.heliyon.2021.e05897

**Published:** 2021-01-21

**Authors:** Tony Lindeberg

**Affiliations:** Computational Brain Science Lab, Division of Computational Science and Technology, KTH Royal Institute of Technology, SE-100 44 Stockholm, Sweden

**Keywords:** Receptive field, Functional model, Gaussian derivative, Scale covariance, Affine covariance, Galilean covariance, Temporal causality, Illumination invariance, Retina, LGN, Primary visual cortex, Simple cell, Double-opponent cell, Vision

## Abstract

This article gives an overview of a normative theory of visual receptive fields. We describe how idealized functional models of early spatial, spatio-chromatic and spatio-temporal receptive fields can be derived in a principled way, based on a set of axioms that reflect structural properties of the environment in combination with assumptions about the internal structure of a vision system to guarantee consistent handling of image representations over multiple spatial and temporal scales. Interestingly, this theory leads to predictions about visual receptive field shapes with qualitatively very good similarities to biological receptive fields measured in the retina, the LGN and the primary visual cortex (V1) of mammals.

## 1 Introduction

**Figure 1:**
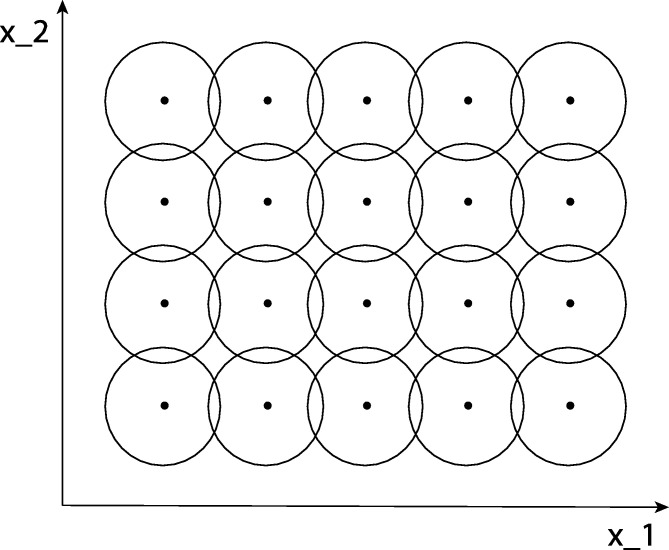
A traditional definition of the notion of a receptive field is as a region in the visual field for which a visual sensor/neuron/operator responds to visual stimuli. In this figure, we have illustrated a set of receptive fields over the spatial domain that partially overlap, and where all the receptive fields have the same size. More generally, we could consider distributions of receptive fields over space or space-time that have varying sizes, shapes and orientations in image space as well as having different directions in joint space-time. Adjacent receptive fields could also have substantially larger relative overlap than displayed here. In this work, we focus on a functional description of such linear receptive fields, concerning how a neuron responds to visual stimuli over image space regarding spatial receptive fields or over joint space-time regarding spatio-temporal receptive fields.

The light distribution that reaches a visual sensor, such as the retina, carries information about the environment to a visual observer. The information necessary to infer properties about the surrounding world from this light distribution is, however, not contained in the measurement of image intensity at any single image point in isolation. Instead, the relevant information is mediated by the *relationships* between image intensities over local neighbourhoods. An underlying reason for this is that the incoming light constitutes an *indirect* source of information that depends on the interaction between geometric and material properties of objects in the surrounding world and on external illumination sources. Another main reason why cues to the environment need to be collected over *regions* in image space as opposed to at single image points is that the measurement process by itself requires the accumulation of energy over non-infinitesimal support regions over space and time. Such a region in the visual field, for which a neuron responds to visual stimuli, is traditionally referred to as a *receptive field* (Hubel and Wiesel [1], [2], [3]) (see Fig. 1).

In this work, we focus on a functional description of receptive fields, regarding how a neuron with a purely spatial receptive field responds to visual stimuli over image space, and regarding how a neuron with a spatio-temporal receptive field responds to visual stimuli over space and time (DeAngelis et al. [4], [5]).

If we consider the theoretical and computational problem of designing a vision system that is going to derive properties of the surrounding world from light reflected from it, we may ask what types of image operations should be performed on the image data. Would it be possible to perform *any* type of image operation, or are there classes of image operations that are more natural or more effective? Specifically, with regard to the notion of receptive fields, we may ask what shapes of receptive field profiles would be reasonable or desirable. Is it possible to express a theory for how receptive fields “ought to” respond to visual data?

From a first inspection, such a problem could possibly be regarded as intractable, unless the prerequisites of the question could be further specified. It does, however, turn out to be possible to address this question in a systematic manner, based on a framework known as *scale-space theory* (Iijima [6]; Witkin [7]; Koenderink [8]; Koenderink and van Doorn [9], [10]; Lindeberg [11], [12], [13], [14]; Florack [15]; Sporring et al. [16]; Weickert et al. [17]; ter Haar Romeny [18]), which has been developed in the area of computer vision. This field has established a paradigm of imposing a set of *structural constraints* on the first stages of visual processing that reflect *symmetry properties* of the environment. From an axiomatic treatment based on such assumptions, it turns out to be possible to restrict the class of permissible image operations substantially.

**Figure 2:**
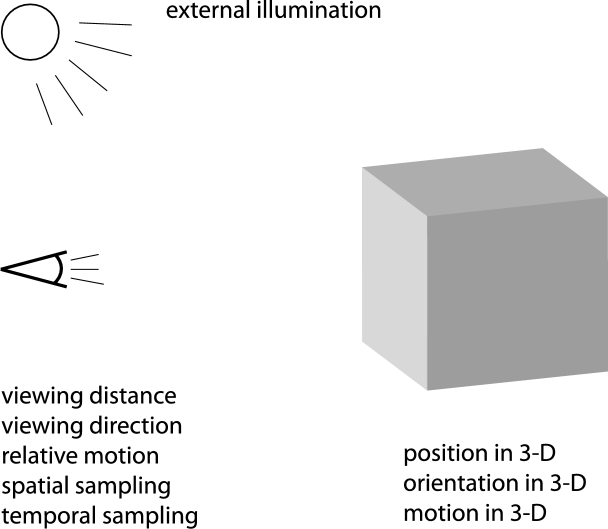
Basic factors that influence the formation of images for an eye with a two-dimensional retina that observes objects in the three-dimensional world. In addition to the position, the orientation and the motion of the object in 3-D, the perspective projection onto the retina is affected by the viewing distance, the viewing direction and the relative motion of the eye in relation to the object, the spatial and the temporal sampling characteristics of the neurons in the retina as well the usually unknown external illumination field in relation to the geometry of the scene and the observer.

The subject of this article is to present a comprehensive overview of a theory for how structural requirements on the first stages of visual processing as formulated based on scale-space theory can be used for deriving idealized functional models of visual receptive fields, and to develop implications of how these theoretical results can be used when modelling receptive fields in the retina, the lateral geniculate nucleus and the primary visual cortex. A main message is that we derive idealized functional models for linear receptive fields *by necessity*, starting from a small set of symmetry requirements that reflect properties of the visual world that one may naturally require an idealized vision system, or a biological organism subject to strong evolutionary pressure, to be adapted to, to enable a consistent handling of receptive field responses in terms of provable covariance or invariance properties under natural image transformations (see Fig. 2).

**Figure 3:**
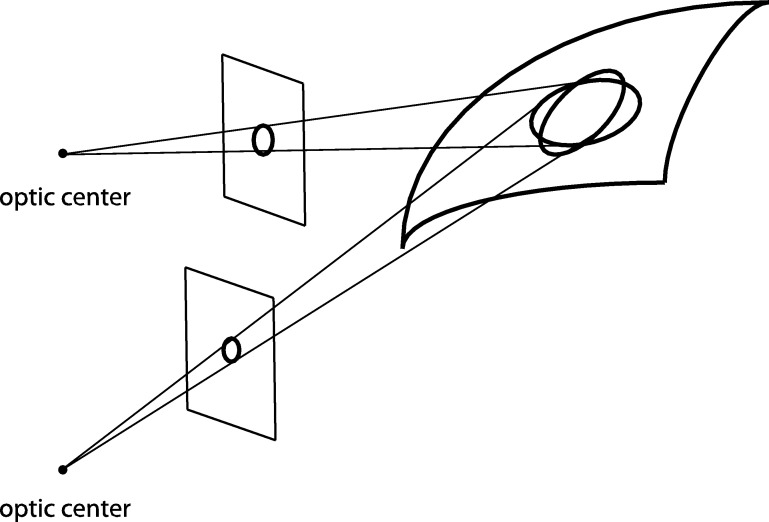
Illustration of the importance of covariance of the receptive field responses under natural image transformations. Consider a vision system that computes image features from image data based on image operations that are formulated over rotationally symmetric support regions in the spatial image domain. If such image measurements are performed for two different viewing directions relative to the same three-dimensional surface patch, then the backprojections of the image operations onto the tangent plane surface of the object will, in general, correspond to different regions in physical space over which corresponding information will be weighted differently. If such image features are in turn to be used for deriving three-dimensional shape cues of the object from binocular cues, such as surface orientation, then there will be a systematic error caused by the mismatch between the backprojections of the receptive fields from the image domain onto the world. By requiring the family of receptive fields to be covariant under local affine image deformations, it is possible to reduce this amount of mismatch, such that the backprojected receptive fields can be made *equal*, when projected onto the tangent plane of the surface by local linearizations of the perspective mapping. In this way, the source to error caused by mismatch between the two different receptive fields is eliminated. Corresponding effects occur when analyzing spatio-temporal image data based on receptive fields that are restricted to being space-time separable only. If an object is observed over time from two observations having different relative motions between the viewing direction and the observer, then the corresponding receptive fields cannot be matched unless the family of receptive fields possesses sufficient covariance properties under local Galilean transformations.

If the receptive field responses do not obey covariance under the basic classes of geometric image transformations, then it will not be possible to match the early image representations between *e.g.* different views of the same scene, which implies that the vision system will perform systematic errors *e.g.* when deriving shape cues from a three-dimensional scene, as illustrated in Fig. 3.

In this respect, the treatment has similarities to the way theories are formulated in theoretical physics, where symmetry properties of the environments constitute key components underlying the formulation of physical theories of the world. The treatment that will follow will be general in the sense that it *encompasses spatial, spatio-chromatic and spatio-temporal receptive fields within the same unified theory*.

Early mathematical necessity results underlying this theory were presented in [13], and earlier versions of this theory in a format for a computational biology audience have been presented in [19], [20]. More recently, a very much improved model for the case of a time-causal spatio-temporal domain was presented in [21], where the future cannot be accessed and the receptive fields have to be solely based on information from the present moment and a compact buffer of the past, written for an audience in the area of mathematical imaging.

That material may, however, be less easy to access for vision researchers in biology, medicine or psychophysics. Specifically, the replacement of certain assumptions (axioms) in [19], [20] with new assumptions (axioms) in [21] may require substantial efforts for readers not previously familiar with this type of theoretical modelling. This has motivated the need for an overview article of the improved theory, intended to be more easy to access, and with a more direct focus on biological implications. Thus, this paper presents an improved axiomatic structure on a compact form more easy to access compared to the original publications and updated with respect to the better time-causal model.

It will be shown that the presented framework leads to predictions of *receptive field profiles* in good agreement with receptive field measurements reported in the literature (Hubel and Wiesel [1], [2], [3]; DeAngelis et al. [4], [5]; Conway and Livingstone [22]; Johnson et al. [23]). Specifically, explicit phenomenological models will be given of neurons in the LGN and simple cells in the primary visual cortex, with comparisons to related models in terms of Gabor functions (Marčelja [24]; Jones and Palmer [25], [26]; Ringach [27], [28]), differences of Gaussians (Rodieck [29]) and Gaussian derivatives (Koenderink and van Doorn [9]; Young [30]; Young et al. [31], [32]). An important consequence of the theory is that the evolution properties of the receptive field profiles can be described by diffusion equations. They are therefore suitable for implementation in a biological architecture, since the computations can be expressed in terms of communications between neighbouring computational units, where either a single computational unit or a group of computational units may be interpreted as corresponding to a neuron or a group of neurons.^1^ Such computational models based on diffusion equations do also arise in mean field theories that approximate the computations that are performed by populations of neurons (Omurtag et al. [33]; Mattia and Guidic [34]; Faugeras et al. [35]).

### 1.1 Structure of this article

This paper is organized as follows: Section 2 gives an overview of and motivations to the assumptions that the theory is based on. A set of structural requirements is formulated to capture the effect of natural image transformations onto the illumination field that reaches the retina and to guarantee internal consistency between image representations that are computed from receptive field responses over multiple spatial and temporal scales.

This set of structural requirements partially overlaps with the structural requirements in [19], [20], while the axiomatic structure has been substantially changed regarding a time-causal temporal domain according to the more recent theory in [21]. This is the most practically relevant case for realistic modelling of biological vision, since there is no way to access the future in a real-time situation, but which is not at all handled in the earlier spatio-temporal modeling work by e.g. Young et al. [31], [32].

Section 3 describes linear receptive families that arise as consequences of these assumptions for the cases of either a purely spatial domain or a joint spatio-temporal domain. The issue of how to perform relative normalization between receptive field responses over multiple spatial and temporal scales is treated, so as to enable comparisons between receptive field responses at different spatial and temporal scales. We also show how the influence of illumination transformations and exposure control mechanisms on the receptive field responses can be handled, by describing invariance properties obtained by applying the derived linear receptive fields over a logarithmically transformed intensity domain.

The consequences of these assumptions for spatial and spatio-temporal domains, which are described in Sections 3.1 and 3.2, constitute more explicit reformulations of results in [19], [20]. These reformulations have additionally been made so that they encompass a time-causal spatio-temporal domain based on results in [21]. The material in Section 3.3 is a new statement of normalization results, partly based a theory for scale selection in [36], while extended from spatially isotropic Gaussian kernels to affine Gaussian kernels, and partially also based on results in [21]. Section 3.4 describes an adaptation of theoretical results in [19] and [37] to this specific domain.

Section 4 shows examples of how spatial, spatio-chromatic and spatio-temporal receptive fields in the retina, the LGN and the primary visual cortex can be well modelled by the derived receptive field families.

Several of these figures are similar to figures in [19], [20] and in [21]. These results have, however, also been cleaned by replacing the previous time-causal models in [19], [20] with the much better time-causal theory in [21], and updating the distribution parameter *c* from the previous default value $c=\sqrt{2}$ to the value c=2 found more suitable for computer vision algorithms that operate on these time-causal spatio-temporal receptive fields with real-time requirements of shorter temporal delays [38].

Section 5 gives relations to previous work, including conceptual and theoretical comparisons to previous use of Gabor models of receptive fields, approaches for learning receptive fields from image data and previous applications of a logarithmic transformation of the image intensities. Finally, Section 6 summarizes some of the main results.

## 2 Assumptions underlying the theory: structural requirements

In the following, we shall describe a set of structural requirements that can be stated concerning: (i) spatial geometry, (ii) spatio-temporal geometry, (iii) the image measurement process with its close relationship to the notion of scale, (iv) internal representations of image data that are to be computed by a general purpose vision system and (v) the parameterization of image intensity with regard to the influence of illumination variations.

For modelling the image formation process, we will at any point in the retina approximate the spherical retina by a perspective projection onto the tangent plane of the retinal surface at that image point, below represented as the image plane. Additionally, we will approximate the possibly non-linear geometric transformations regarding spatial and spatio-temporal geometry by local linearizations at every image point, and corresponding to the derivatives of the possibly non-linear transformations. In these ways, the theoretical analysis can be substantially simplified, while still enabling accurate modelling of essential functional properties of receptive fields in relation to the effects of natural image transformations as arising from interactions with the environment.

**Figure 4:**
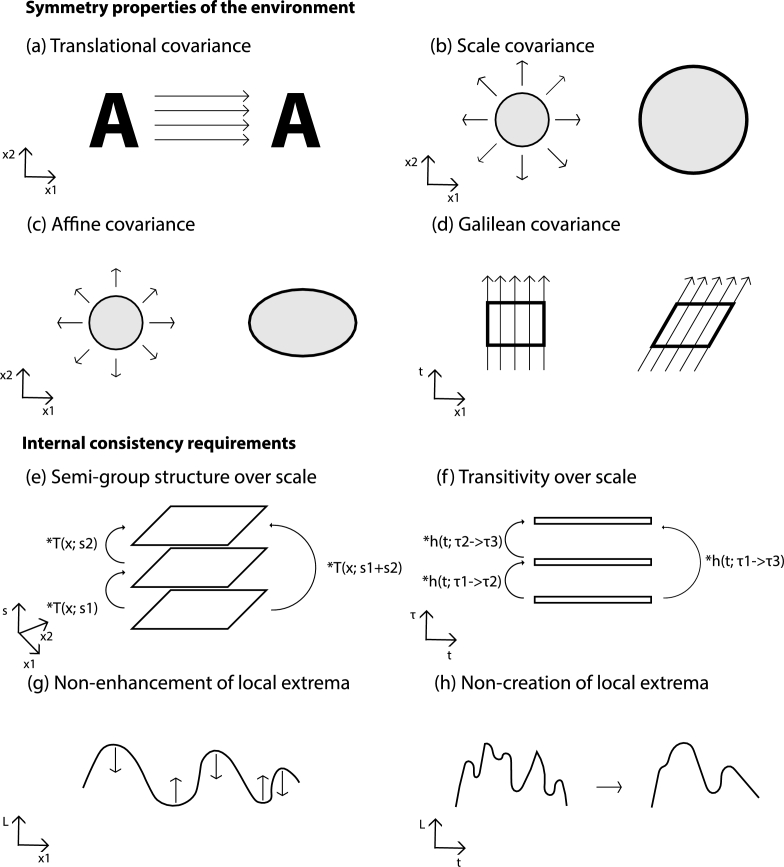
Schematic illustration of main assumptions underlying the proposed normative theory for visual receptive fields, regarding (i) transformation properties of the environment and (ii) internal consistency requirements to guarantee internally consistent image representations over multiple spatial and temporal scales. (a) *Translational covariance* means that visual representations of objects should be processed in a similar manner over the entire visual field. (b) *Scale covariance* means that scaling transformations, as occur in the visual domain because of objects of different size and objects at different distances to the observer, should be processed in a similar manner such that the receptive field responses can be matched. (c) *Affine covariance* is a generalization of scale covariance to non-uniform scaling transformations, as occur when surface structures are foreshortened for surfaces with a non-frontal slant angle relative to the tangent plane of the surface. (d) *Galilean covariance* means that if we observe objects or events that move relative to a fixed viewing direction, then these visual patterns should be processed in a conceptually similar way as if we observe the same patterns with the gaze direction following the same objects or events, and in such a way that the two types of spatio-temporal image representations can be matched. (e) The assumption of a *semi-group structure over spatial scales* implies that with a spatial smoothing operation in terms of convolution operations, which follows from a combination of the assumptions of translational covariance and linearity, the composition of two spatial smoothing operations with scale parameters *s*_1_ and *s*_2_ should be a spatial smoothing operation of a similar form and with added values of scale parameters *s*_1_ + *s*_2_. (f) The assumption of a *transitivity structure over temporal scales* implies that the composition of two temporal smoothing operations from temporal scales *τ*_1_ to *τ*_2_ and from temporal scales *τ*_2_ to *τ*_3_ should be a similar type of temporal smoothing operation from temporal scales *τ*_1_ to *τ*_3_ (while without imposing an additive structure of the temporal scale parameters). (g) The assumption of *non-enhancement of local extrema* means that the spatial smoothing operation that determines the shape of the spatial receptive fields should obey the property that the smoothed intensity value *L* at a spatial maximum must not increase with increasing scale and that the intensity value at a spatial minimum must not decrease with increasing scale. (h) The assumption of *non-creation of local extrema* implies that the temporal smoothing operation that determines the temporal shape of the spatio-temporal receptive fields must not increase the number of local extrema in a purely temporal signal.

By necessity, some parts of the presentation in this section will be somewhat technical, if we want to clearly mathematically define the assumptions that the theory rests upon. For the hasty reader, who may be more interested in the implications of the theory, we have made a schematic summary of most of the main assumptions in Fig. 4. After getting an overview of these assumptions from this figure, the hasty reader may then proceed to Section 3, where it is shown how these assumptions lead to idealized families of visual receptive fields, then backtracking to this section again if necessary.

### 2.1 Static image data over a spatial domain

In the following, we will describe a theoretical model for the computational function of applying visual receptive fields to local image patterns.

For time-independent data *f* over a two-dimensional spatial image domain, we would like to define a family of image representations L(⋅;s) over a possibly multi-dimensional scale parameter *s*, where the internal image representations L(⋅;s) are computed by applying some parameterized family of image operators $T_{s}$ to the image data *f*:

(1) $$L(\cdot ;\;s)=T_{s}\;f(\cdot ).$$

Specifically, we will assume that the family of image operators $T_{s}$ should satisfy:

*a) Linearity.* For the earliest processing stages to have as low risk as possible of making irreversible decisions that may affect later processing stages, we assume that the first layers of receptive fields should be linear

(2) $$T_{s}(a_{1}f_{1}+a_{2}f_{2})=a_{1}T_{s}f_{1}+b_{1}T_{s}f_{2}.$$

Specifically, linearity implies that any particular scale-space properties (to be detailed below) that we derive for the zero-order image representation *L* will transfer to any spatial derivative $L_{x_{1}^{\alpha_{1}}x_{2}^{\alpha_{2}}}$ of *L*, so that

(3) $$L_{x_{1}^{\alpha_{1}}x_{2}^{\alpha_{2}}}(\cdot ;\;s)=\partial_{x_{1}^{\alpha_{1}}x_{2}^{\alpha_{2}}}(T_{s}\;f(\cdot ))=T_{s}(\partial_{x_{1}^{\alpha_{1}}x_{2}^{\alpha_{2}}}f(\cdot ))$$

where $\alpha_{1}$ and $\alpha_{2}$ are the derivative orders for the two spatial dimensions $x_{1}$ and $x_{2}$.

This means that different types of image structures, irrespective of what order of spatial differentiation they respond mostly to, will be treated in a structurally similar manner. In this way, we reduce the risk that the first layers of visual receptive fields could make early decisions dedicated to certain types of image structures that later processing stages could then not later recover from.

In this sense, the assumption of linearity reflects the requirement of a lack of bias to particular types of image structures, with the underlying aim that the processing performed in the first processing stages should be *generic*, to be used as input for a large variety of visual tasks. By the assumption of linearity, local image structures that are captured by *e.g.* first- or second-order derivatives will be treated in a structurally similar manner, which would not necessarily be the case if the first local neighbourhood processing stage of the first layer of receptive fields would instead be allowed to be genuinely non-linear.^2^

This genericity property is closely related to the basic property of the mammalian vision system, that the computations performed in the retina^3^, the LGN^4^ and the primary visual cortex provide general purpose output that is used as input to higher-level visual areas.

*b) Translational covariance.* To ensure that the visual interpretation of an object should be the same irrespective of its position in the image plane, we assume that the first processing stages should be covariant under translations, so that if an object is moved a distance $\Delta x=(\Delta x_{1},\Delta x_{2})$ in the image plane, the receptive field response should remain on a similar form, while shifted with the same distance. Formally, this requirement can be stated that the family of image operators $T_{s}$ should commute with the shift operator defined by $S_{\Delta x}(f)(x)=f(x-\Delta x)$:

(4) $$T_{s}\left(S_{\Delta x}f\right)=S_{\Delta x}\left(T_{s}f\right).$$

In other words, if we shift the input by a translation and then apply the receptive field operator $T_{s}$, the result should be similar as applying the receptive field operator to the original input and then shifting the result.

*c) Convolution structure.* Together, the assumptions about linearity and translational covariance imply that $T_{s}$ will correspond to a convolution operator [54]. This implies that the representation *L* can be computed from the image data *f* by convolution with some parameterized family of convolution kernels T(⋅;s):

(5) L(⋅;s)=T(⋅;s)⁎f(⋅).

*d) Semi-group structure over spatial scales.* To ensure that the transformation from any finer scale $s_{1}$ to any coarser scale $s_{1}+s_{2}$ should be of the same form for any $s_{2}>0$ (a requirement of algebraic closedness), we assume that the result of convolving two kernels $T(\cdot ;\;s_{1})$ and $T(\cdot ;\;s_{2})$ from the family with each other should be a kernel within the same family of kernels and with added parameter values $T(\cdot ;\;s_{1}+s_{2})$:

(6) $$T(\cdot ;\;s_{1})⁎T(\cdot ;\;s_{2})=T(\cdot ;\;s_{1}+s_{2}).$$

This assumption specifically implies that the representation $L(\cdot ;\;s_{2})$ at a coarse scale $s_{2}$ can be computed from the representation $L(\cdot ;\;s_{1})$ at a finer scale $s_{1}<s_{2}$ by a convolution operation of the same form (5) as the transformation from the original image data *f*, while using the difference in scale levels $s_{2}-s_{1}$ as the parameter

(7) $$L(\cdot ;\;s_{2})=T(\cdot ;\;s_{2}-s_{1})⁎L(\cdot ;\;s_{1}).$$

This property does in turn imply that if we are able to derive specific properties of the family of transformations $T_{s}$ (to be detailed below), then these properties will not only hold for the transformation from the original image data *f* to the representations L(⋅;s) at coarser scales, but also between any pair of scale levels $s_{2}>s_{1}$, with the aim that image representations at coarser scales should be regarded as simplifications of corresponding image representations at finer scales.

In terms of mathematical concepts, this form of algebraic structure is referred to as a semi-group structure over spatial scales

(8) $$T_{s_{1}}T_{s_{2}}=T_{s_{1}+s_{2}}.$$

*e) Scale covariance under spatial scaling transformations.* If a visual observer looks at the same object from different distances, we would like the internal scale-space representations derived from the receptive field responses to be sufficiently similar, so that the object can be recognized as the same object, while appearing with a different size on the retina. Specifically, it is thereby natural to require that the receptive field responses should be of a similar form, while resized in the image plane.

This corresponds to a requirement of spatial scale covariance under uniform scaling transformations of the spatial domain $x^{\prime }=S_{s}\;x$:

(9) $$L^{\prime }(x^{\prime };\;s^{\prime })=L(x;\;s)\;\Leftrightarrow \;T_{S_{s}(s)}\;S_{s}\;f=S_{s}\;T_{s}\;f$$

to hold for some transformation $s^{\prime }=S_{s}(s)$ of the scale parameter *s*.

*f) Affine covariance under spatial affine transformations.* If a visual observer looks at the same local surface patch from two different viewing directions, then the local surface patch may be deformed in different ways onto the different views and with different amounts of perspective foreshortening from the different viewing directions. If we approximate the local deformations caused by the perspective mapping by local affine transformations, then the transformation between the two differently deformed views of the local surface patch can in turn be described by a composed local affine transformation $x^{\prime }=A\;x$.

If we are to use receptive field responses as a basis for higher level visual operations, it is natural to require that the receptive field response of an affine deformed image patch should remain on a similar form, while being reshaped by a corresponding affine transformation.

This corresponds to a requirement of affine covariance under general affine transformations $x^{\prime }=A\;x$:

(10) $$L^{\prime }(x^{\prime };\;s^{\prime })=L(x;\;s)\;\Leftrightarrow \;T_{A(s)}\;A\;f=A\;T_{s}\;f$$

to hold for some transformation $s^{\prime }=A(s)$ of the scale parameter.

*g) Non-creation of new structure with increasing scale.* If we apply the family of transformations $T_{s}$ for computing representations at coarser scales from representations at finer scales according to (1) and (7), there could be a potential risk that the family of transformations could amplify spurious structures in the input to produce macroscopic amplifications in the representations at coarser scales that do not directly correspond to simplifications of corresponding structures in the original image data. To prevent such undesirable phenomena from occurring, we require that local spurious structures must not be amplified and express this condition in terms of the evolution properties over scales at local maxima and minima in the image intensities as smoothed by the family of convolution kernels T(⋅;s): If a point $x_{0}$ for some scale $s_{0}$ is a local maximum point in the image plane, then the value at this maximum point $L(x_{0};\;s_{0})$ must not increase to coarser scales $s>s_{0}$. Similarly, if a point is a local minimum point in the image plane, then the value at this minimum point $L(x_{0};\;s_{0})$ must not decrease to coarser scales $s>s_{0}$.

**Figure 5:**
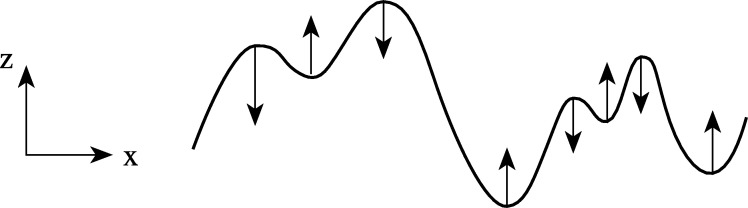
Illustration of the notion of non-enhancement of local extrema, which is a way to restrict the class of possible image operations by preventing new structures from being created from finer to coarser levels of scales. Non-enhancement of local extrema means that the value at a local maximum must not increase and that the value at a local minimum must not decrease with increasing scale *s*.

Formally, this requirement that new structures should not be created from finer to coarser scales can be formalized into the requirement of *non-enhancement of local extrema*, which implies that if at some scale $s_{0}$ a point $x_{0}$ is a local maximum (minimum) for the mapping from *x* to $L(x;\;s_{0})$, then (see Fig. 5):

• $(\partial_{s}L)(x;\;s)\leq 0$ at any spatial maximum,
• $(\partial_{s}L)(x;\;s)\geq 0$ at any spatial minimum.

This condition implies a strong condition on the class of possible smoothing kernels T(⋅;s).

### 2.2 Time-dependent image data over joint space-time

To model the computational function of spatio-temporal receptive fields in time-dependent image patterns, we do for a time-dependent spatio-temporal domain first inherit the structural requirements regarding a spatial domain and complement the spatial scale parameter *s* by a temporal scale parameter *τ*. In addition, we assume:

*a) Scale covariance under temporal scaling transformations.* If a similar type of spatio-temporal event f(x,t) occurs at different speeds, faster or slower, it is natural to require that the receptive field responses should be of a similar form, while occurring correspondingly faster or slower.

This corresponds to a requirement of temporal scale covariance under a temporal scaling transformation of the temporal domain $t^{\prime }=S_{\tau }t$:

(11) $$L^{\prime }(x^{\prime },t^{\prime };\;s^{\prime },\tau^{\prime })=L(x,t;\;s,\tau )\;\Leftrightarrow \;T_{S_{\tau }(s,\tau )}\;S_{\tau }\;f=S_{\tau }\;T_{s,\tau }\;f$$

to hold for some transformation $\left(s^{\prime },\tau^{\prime })=S_{\tau }(s,\tau \right)$ of the spatio-temporal scale parameters (s,τ).

*b) Galilean covariance under Galilean transformations.* If an observer looks at the same object in the world for different relative motions $v=(v_{1},v_{2})$ between the object and the observer, it is natural to require that the internal scale-space representations of the object should be sufficiently similar, so as to enable a coherent perception of the object under different relative motions relative to the observer. Specifically, we may require that the receptive field responses under relative motions should remain on the same form, while being transformed in a corresponding way as the relative motion pattern.

If we at any point in space-time locally linearize the possibly non-linear motion pattern $x(t)=(x_{1}(t),x_{2}(t))$ by a local Galilean transformation $x^{\prime }=x+v\;t$ over space-time

(12) $$f^{\prime }=G_{v}\;f\;\Leftrightarrow \;f^{\prime }(x^{\prime },t^{\prime })=f(x,t)\;\text{with}\;x^{\prime }=x+v\;t,$$

then the requirement of guaranteeing a consistent visual interpretation under different relative motions between the object and the observer can be stated as a requirement of Galilean covariance:

(13) $$L^{\prime }(x^{\prime },t^{\prime };\;s^{\prime },\tau^{\prime })=L(x,t;\;s,\tau )\;\Leftrightarrow \;T_{G_{v}(s,\tau )}\;G_{v}\;f=G_{v}\;T_{s,\tau }\;f$$

to hold for some transformation $G_{v}(s,\tau )$ of the spatio-temporal scale parameters (s,τ).

*c) Semi-group structure over temporal scales in the case of a non-causal temporal domain.* To ensure that the image representations between different spatio-temporal scale levels $\left(s_{1},\tau_{1}\right)$ and $\left(s_{2},\tau_{2}\right)$ should be sufficiently well-behaved internally, we will make use of different types of assumptions depending on whether the temporal domain is regarded as time-causal or non-causal. Over a time-causal temporal domain, the future cannot be accessed, which is the basic condition for real-time visual perception by a biological organism. Over a non-causal temporal domain, the temporal kernels may extend to the relative future in relation to any pre-recorded time moment, which is sometimes used as a conceptual simplification when analysing pre-recorded time-dependent data, although not at all realistic in a real-world setting.

For the case of a non-causal temporal domain, we make use of a similar type of semi-group property (8) as formulated over a purely spatial domain, while extending the semi-group property over both the spatial scale parameter *s* and the temporal scale parameter *τ*:

(14) $$T_{s_{1},\tau_{1}}T_{s_{2},\tau_{2}}=T_{s_{1}+s_{2},\tau_{1}+\tau_{2}}.$$

In analogy with the case of a purely spatial domain, this requirement guarantees that the transformation from any finer spatio-temporal scale level $\left(s_{1},\tau_{1}\right)$ to any coarser spatio-temporal scale level $\left(s_{2},\tau_{2})\geq (s_{1},\tau_{1}\right)$ will always be of the same form (algebraic closedness)

(15) $$L(\cdot ,\cdot ;\;s_{2},\tau_{2})=T_{s_{2}-s_{1},\tau_{2}-\tau_{1}}\;L(\cdot ,\cdot ;\;s_{1},\tau_{1}).$$

Specifically, this assumption implies that if we are able to establish desirable properties of the family of transformations $T_{s,\tau }$ (to be detailed below), then these relations hold between any pair of spatio-temporal scale levels $\left(s_{1},\tau_{1}\right)$ and $\left(s_{2},\tau_{2}\right)$ with $\left(s_{2},\tau_{2})\geq (s_{1},\tau_{1}\right)$.

*d) Cascade structure over temporal scales in the case of a time-causal temporal domain.* Since it can be shown that the assumption of a semi-group structure over temporal scales leads to undesirable temporal dynamics in terms of *e.g.* longer temporal delays for a time-causal temporal domain [55, Appendix A], we do for a time-causal temporal domain instead assume a weaker cascade smoothing property over temporal scales for the temporal smoothing kernel over temporal scales

(16) $$L(\cdot ;\;\tau_{2})=h(\cdot ;\;\tau_{1}\mapsto \tau_{2})⁎L(\cdot ;\;\tau_{1}),$$

where the temporal kernels h(t;τ) should for any triplets of temporal scale values and temporal delays $\tau_{1}$, $\tau_{2}$ and $\tau_{3}$ obey the transitive property

(17) $$h(\cdot ;\;\tau_{1}\mapsto \tau_{2})⁎h(\cdot ;\;\tau_{2}\mapsto \tau_{3})=h(\cdot ;\;\tau_{1}\mapsto \tau_{3}).$$

This weaker assumption of a cascade smoothing property (16) still ensures that an image representation at a coarser temporal scale $\tau_{2}$ should with a corresponding requirement of an accompanying simplifying condition on the family of kernels *h* (to be detailed below) constitute a simplification of the representation at a finer temporal scale $\tau_{1}$, while not implying as hard constraints as a semi-group structure.

*e) Non-enhancement of local space-time extrema in the case of a non-causal temporal domain.* In the case of a non-causal temporal domain, we again build on the notion of non-enhancement of local extrema to guarantee that the image representations at coarser spatio-temporal scales should constitute true simplifications of corresponding representations at finer scales. Over a spatio-temporal domain, we do, however, state the requirement in terms of local extrema over joint space-time instead of over local extrema over image space. If a point $\left(x_{0},t_{0}\right)$ for some scale $\left(s_{0},\tau_{0}\right)$ is a local maximum point over space-time, then the value at this maximum point $L(x_{0},t_{0};\;s_{0},\tau_{0})$ must not increase to coarser spatio-temporal scales $\left(s,\tau )\geq (s_{0},\tau_{0}\right)$. Similarly, if a point is a local minimum point over space-time, then the value at this minimum point $L(x_{0},t_{0};\;s_{0},\tau_{0})$ must not decrease to coarser spatio-temporal scales $\left(s,\tau )\geq (s_{0},\tau_{0}\right)$.

Formally, this requirement of non-creation of new structure from finer to coarser spatio-temporal scales can be stated as follows: If at some scale $\left(s_{0},\tau_{0}\right)$ a point $\left(x_{0},t_{0}\right)$ is a local maximum (minimum) for the mapping from (x,t) to $L(x,t;\;s_{0},\tau_{0})$, then

• $\alpha \;(\partial_{s}L)(x,t;\;s,\tau )+\beta \;(\partial_{\tau }L)(x,t;\;s,\tau )\leq 0$ at any spatio-temporal maximum
• $\alpha \;(\partial_{s}L)(x,t;\;s,\tau )+\beta \;(\partial_{\tau }L)(x,t;\;s,\tau )\geq 0$ at any spatio-temporal minimum

should hold in any positive spatio-temporal direction defined from any non-negative linear combinations of *α* and *β*. This condition implies a strong condition on the class of possible smoothing kernels T(⋅,⋅;s,τ).

*f) Non-creation of new local extrema or zero-crossings for a purely temporal signal in the case of a time-causal temporal domain.* In the case of a time-causal temporal domain, we do instead state a requirement for purely temporal signals, based on the cascade smoothing property (16). We require that for a purely temporal signal f(t), the transformation from a finer temporal scale $\tau_{1}$ to a coarser temporal scale $\tau_{2}$ must not increase the number of local extrema or the number of zero-crossings in the signal.

## 3 Idealized receptive field families

### 3.1 Spatial image domain

Based on the above assumptions in Section 2.1, it can be shown [13] that when complemented with certain regularity assumptions in terms of Sobolev norms, they imply^5^ that a spatial scale-space representation *L* as determined by these assumptions must satisfy a diffusion equation of the form^6^

(18) $$\partial_{s}L=\frac{1}{2}\;\nabla^{T}\left(\Sigma \;\nabla L\right)-\delta^{T}\nabla L$$

for some symmetric positive definite covariance matrix $\Sigma =\left(\begin{array}{cc} \Sigma_{11}\; & \Sigma_{12}\\ \Sigma_{12}\; & \Sigma_{22} \end{array}\right)$ and some translation vector $\delta ={\left(\delta_{1},\delta_{2}\right)}^{T}$, where ∇ denotes the (vertical) spatial gradient operator and ^*T*^ its transpose such that $\nabla^{T}=(\partial_{x_{1}},\partial_{x_{2}})$.

Expanding the matrix and vector notation to elements, this equation can also be written

(19) $$\partial_{s}L=\frac{1}{2}\;(\Sigma_{11}L_{x_{1}x_{1}}+2\Sigma_{12}L_{x_{1}x_{2}}+\Sigma_{22}L_{x_{2}x_{2}})-\delta_{1}L_{x_{1}}-\delta_{2}L_{x_{2}},$$

where the subscripts with respect to *s*, $x_{1}$ and $x_{2}$ denote derivatives with respect to these variables.

This expression is physically analogous to a diffusion equation that describes how a heat distribution corresponding to the image intensities *L* evolves as function of time in an inhomogeneous medium with spatial scale *s* here taking the role of time, with the intensities of the input image *f* as initial condition $L(x_{1},x_{2};\;s)=f(x_{1},x_{2})$ for s=0. The first term, that depends upon Σ, describes how the image intensity *L* diffuses as the scale parameter *s* increases, as function of the anisotropic heat conductivity Σ, which determines how the image intensities may diffuse differently in different spatial directions. The second term, that depends upon *δ*, describes how the image intensities are translated to other spatial positions as function of a spatial drift velocity *δ*.

The first effect results in a smoothing effect that may be different in different spatial directions as determined by the anisotropic covariance matrix Σ. With regard to spatial image transformations, variations of the scale parameter *s* lead to solutions that obey the assumption of scale covariance, to handle objects of different sizes in the world and objects at different distances to the observer.

More general variations of the shape of the covariance matrix Σ do additionally allow for affine covariance, to enable matching of objects that are viewed from different viewing directions relative to the local tangent plane of a smooth surface.

The second translation effect, as determined by the translation vector *δ*, is relevant for handling image disparities between binocular eyes or, for time-dependent images, image structures that move as function of time.

In terms of convolution kernels, the solution of (18) corresponds to convolution with Gaussian kernels of the form

(20) $$g(x;\;\Sigma_{s},\delta_{s})=\frac{1}{2\pi \sqrt{\det\Sigma_{s}}}\;e^{-{\left(x-\delta_{s}\right)}^{T}\Sigma_{s}^{-1}(x-\delta_{s})/2},$$

which for a given $\Sigma_{s}=s\;\Sigma$ and a given $\delta_{s}=s\;\delta$ satisfy (18) (see Appendix A in the supplement for an explicit proof of the property that the family of internal spatial scale-space representations *L* generated by convolution with kernels of the form (20) satisfies the diffusion equation (18)).

If we additionally require these kernels to be mirror symmetric through the origin, then we obtain *affine Gaussian kernels*

(21) $$g(x;\;\Sigma )=\frac{1}{2\pi \sqrt{\det\Sigma }}\;e^{-x^{T}\Sigma^{-1}x/2}.$$

Their spatial derivatives constitute a canonical family for expressing receptive fields over a spatial domain that can be summarized and reparameterized on the form

(22) $$T(x;\;s,\Sigma )=g(x;\;s\;\Sigma )=\frac{1}{2\pi s\sqrt{\det\Sigma }}\;e^{-x^{T}\Sigma^{-1}x/2s},$$

where we have separated the parameters into two components; a scalar scale parameter *s* that represents the size of the Gaussian, *i.e.*, how large it is in the image domain, and a matrix Σ that determines its shape, *i.e.*, how eccentric it is (the ratio between the sizes in the perpendicular principal directions for an anisotropic Gaussian) and the orientation of the main principal axis in the image domain.

The spatial scale-space representations that are obtained by convolution with kernels of this form obey (i) spatial scale covariance as described in Appendix C in the supplement and illustrated in Fig. 21 in the supplement and (ii) spatial affine covariance as described in Appendix D in the supplement and illustrated in Fig. 22 in the supplement.

Incorporating the fact that spatial derivatives of the kernels (22) are also compatible with the assumptions underlying this theory, this does specifically for the case of a two-dimensional spatial image domain lead to spatial receptive fields that can be compactly summarized on the form

(23) $$T_{\varphi^{m_{1}}⊥\varphi^{m_{2}}}(x_{1},x_{2};\;s,\Sigma )=\partial_{\varphi }^{m_{1}}\partial_{⊥\varphi }^{m_{2}}\left(g(x_{1},x_{2};\;s\Sigma )\right),$$

where

• $x=(x_{1},x_{2})$ denotes the spatial coordinates,
• *s* denotes the spatial scale^7^ in units of $s=\sigma^{2}$, where *σ* has dimension [length] and corresponds to the standard deviation of the Gaussian kernel for an isotropic covariance matrix with $\Sigma =I=\left(\begin{array}{cc} 1\; & 0\\ 0\; & 1 \end{array}\right)$,
• Σ denotes a spatial covariance matrix determining the shape of a spatial affine Gaussian kernel (this covariance matrix is assumed to be symmetric positive definite such that $x^{T}\Sigma \;x=\Sigma_{11}\;x_{1}^{2}+2\Sigma_{12}\;x_{1}\;x_{2}+\Sigma_{22}\;x_{2}^{2}>0$ for any $x={\left(x_{1},x_{2}\right)}^{T}\neq 0$),
• $\partial_{\varphi }=\cos\varphi \;\partial_{x_{1}}+\sin\varphi \;\partial_{x_{2}}$ and $\partial_{⊥\varphi }=\sin\varphi \;\partial_{x_{1}}-\cos\varphi \;\partial_{x_{2}}$ denote spatial directional derivative operators in two orthogonal directions *φ* and ⊥*φ* aligned with the eigenvectors of the covariance matrix Σ, where $\partial_{x_{1}}$ and $\partial_{x_{2}}$ denote differentiation with respect the spatial coordinates $x_{1}$ and $x_{2}$,
• $m_{1}$ and $m_{2}$ denote orders of spatial differentiation in the spatial direction *φ* and its orthogonal direction ⊥*φ*, respectively,
• $g(x;\;s\;\Sigma )=\frac{1}{2\pi s\sqrt{\det\Sigma }}e^{-x^{T}\Sigma^{-1}x/2s}$ is an affine Gaussian kernel with its size determined by the spatial scale parameter *s* and its shape by the spatial covariance matrix Σ.

**Figure 6:**
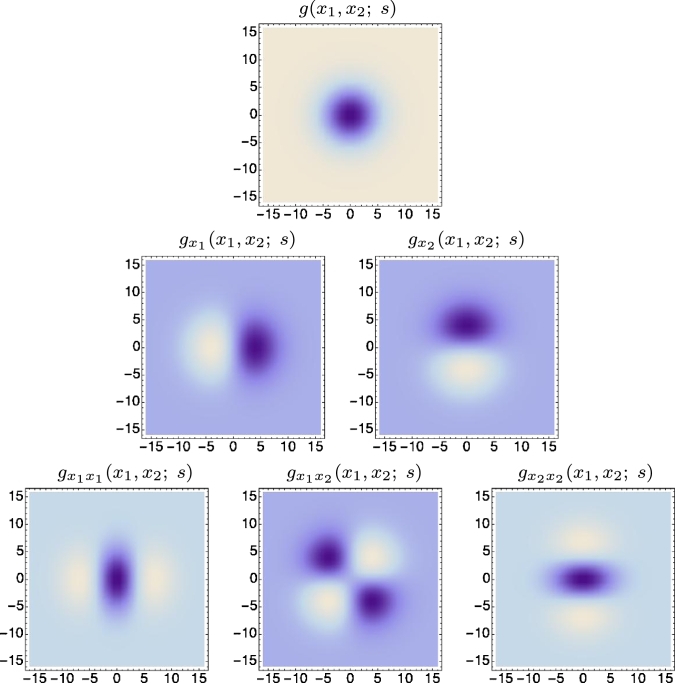
Illustrations of spatial receptive fields formed by the 2-D rotationally symmetric Gaussian kernel (for *s* = 16) and its partial derivatives up to order two. The resulting receptive fields are closed under translations, rotations and scaling transformations. This means that if an image is transformed in these ways, then it will always be possible to find some possibly other receptive field such that the receptive field responses of the original image and the transformed image can be perfectly matched.

**Figure 7:**
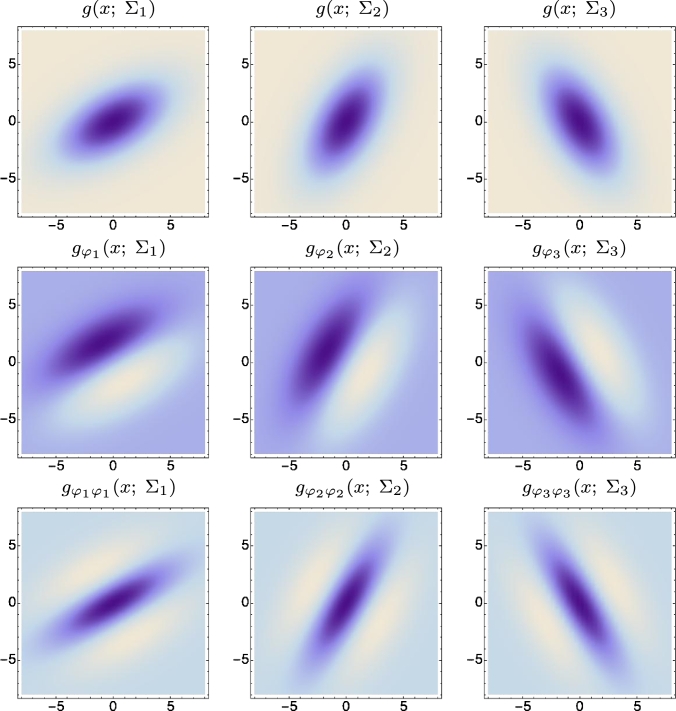
Illustrations of spatial receptive fields formed by affine Gaussian kernels and directional derivatives of these up to order two of, here visualized for three different covariance matrices Σ_1_, Σ_2_ and Σ_3_ that correspond to the major eigendirections *θ*_1_ = *π*/6, *θ*_2_ = *π*/3 and *θ*_3_ = 2*π*/3 of the covariance matrix and with directional derivatives computed in the corresponding orthogonal directions *φ*_1_, *φ*_2_ and *φ*_3_. The resulting family of receptive fields is closed under general affine transformations of the spatial domain, including translations, rotations, scaling transformations and perspective foreshortening. In this figure, however, only variabilities in the orientation of the filter are illustrated, thereby disregarding variabilities in both the size and the degree of elongation. This closedness property implies that receptive field responses computed from different views of a smooth local surface patch can be perfectly matched, if the transformation between the two views can be modelled as a local affine transformation. (Scale parameters *s*_1_ = 16 and *s*_2_ = 4 in the orthogonal eigendirections of the spatial covariance matrices Σ_*i*_.)

Fig. 6 and Fig. 7 show examples of spatial receptive fields from this family up to second order of spatial differentiation. Fig. 6 shows partial derivatives of the Gaussian kernel for the specific case when the covariance matrix Σ is restricted to a unit matrix and the Gaussian kernel thereby becomes rotationally symmetric. The resulting family of receptive fields is closed under scaling transformations over the spatial domain, implying that if an object is seen from different distances to the observer, then it will always be possible to find a transformation of the scale parameter *s* between the two image domains such that the receptive field responses computed from the two image domains can be matched. Fig. 7 shows examples of affine Gaussian receptive fields for covariance matrices Σ that do not correspond to rescaled copies of the unit matrix. The resulting full family of affine Gaussian derivative kernels is closed under general affine transformations, implying that for two different perspective views of a local smooth surface patch, it will always be possible to find a transformation of the covariance matrices Σ between the two domains so that the receptive field responses can be matched, if the transformation between the two image domains is approximated by a local affine transformation.

**Figure 8:**
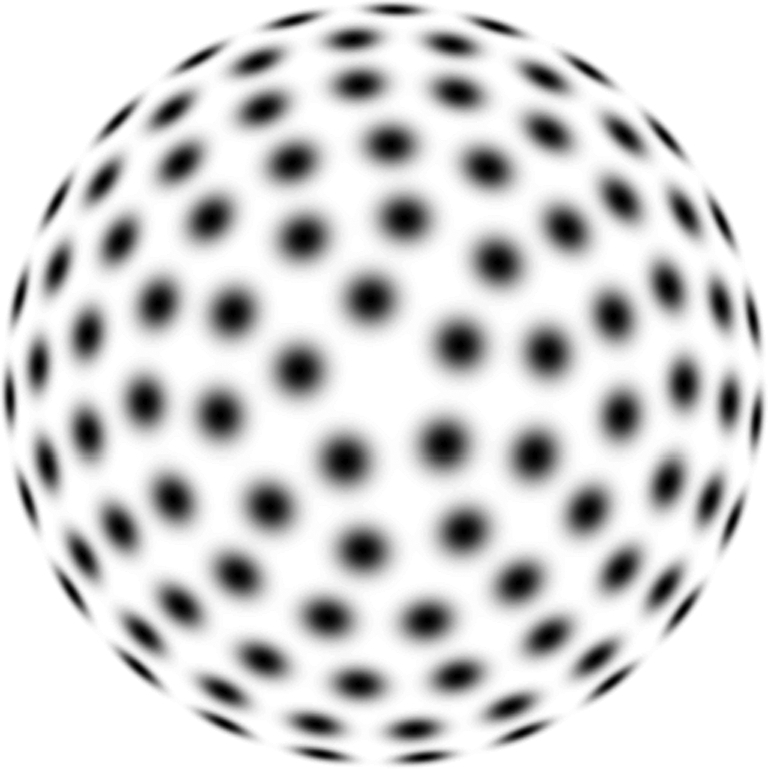
Illustration of the variability of zero-order affine Gaussian receptive fields for a uniform distribution on a hemisphere. In the most idealized version of the theory, one can think of all affine receptive fields with their directional derivatives in preferred directions aligned to the eigendirections of the covariance matrix Σ as being present at any position in the image domain. This variability makes it possible to perfectly match the first-order variability of receptive field responses under variations of the slant and tilt directions of a smooth surface patch.

In the most idealized version of the theory, one should think of receptive fields for all combinations of filter parameters as being present at every image point, as illustrated in Fig. 8 concerning affine Gaussian receptive fields over different orientations in image space and different eccentricities.

### 3.2 Spatio-temporal image domain

Over a non-causal spatio-temporal domain, corresponding arguments as in Section 3.1 lead to a similar form of diffusion equation as in Equation (18), while expressed over the joint space-time domain p=(x,t). After splitting the composed affine Gaussian spatio-temporal smoothing kernel corresponding to (20), while expressed over the joint space-time domain, into separate smoothing operations over space and time, this leads to zero-order spatio-temporal receptive fields of the form [13], [19] (see Appendix B.1 in the supplement for an overview of the logical steps in the derivation that lead to this result):

(24) $$T(x_{1},x_{2},t;\;s,\tau ;\;v,\Sigma )=g(x_{1}-v_{1}t,x_{2}-v_{2}t;\;s\;\Sigma )\;h(t;\;\tau ),$$

where the temporal smoothing kernel h(t;τ) is a one-dimensional Gaussian kernel. After combining that result with the results from corresponding theoretical analysis for a time-causal spatio-temporal domain in [13], [21] (see Appendix B.2 in the supplement for an overview of the logical steps behind this construction), we are lead to a similar form of spatio-temporal smoothing operation, while then using a temporal smoothing kernel h(t;τ) that corresponds to a set of truncated exponential kernels coupled in cascade.

The resulting spatio-temporal scale-space representation obeys (i) spatial scale covariance as described in Appendix E in the supplement and illustrated in Fig. 23 in the supplement, (ii) spatial affine covariance as described in Appendix F in the supplement and illustrated in Fig. 24 in the supplement, (iii) Galilean covariance as described in Appendix G in the supplement and illustrated in Fig. 25 in the supplement and (iv) temporal scale covariance as described in Appendix H in the supplement and illustrated in Fig. 26 in the supplement.

After noting that spatial, temporal and spatio-temporal derivatives of the spatio-temporal smoothing kernels (24) are also compatible with the assumptions because of the linearity assumption, the resulting spatio-temporal derivative kernels constituting the spatio-temporal extension of the spatial receptive field model (23) can be reparameterized and summarized on the following form (see [13], [19], [20], [21]):

(25) $$T_{\varphi^{m_{1}}⊥\varphi^{m_{2}}{\overset{¯}{t}}^{n}}(x_{1},x_{2},t;\;s,\tau ;\;v,\Sigma )=\partial_{\varphi }^{m_{1}}\partial_{⊥\varphi }^{m_{2}}\partial_{\overset{¯}{t}}^{n}\left(g(x_{1}-v_{1}t,x_{2}-v_{2}t;\;s\;\Sigma )\;h(t;\;\tau )\right),$$

where

• $x=(x_{1},x_{2})$ denotes the spatial coordinates,
• *t* denotes time,
• *s* denotes the spatial scale (in dimension of ${\left[\text{length}\right]}^{2}$),
• *τ* denotes the temporal scale (in dimension of ${\left[\text{time}\right]}^{2}$),
• $v={\left(v_{1},v_{2}\right)}^{T}$ denotes a local image velocity,
• Σ denotes a spatial covariance matrix determining the shape of a spatial affine Gaussian kernel,
• $\partial_{\varphi }=\cos\varphi \;\partial_{x_{1}}+\sin\varphi \;\partial_{x_{2}}$ and $\partial_{⊥\varphi }=\sin\varphi \;\partial_{x_{1}}-\cos\varphi \;\partial_{x_{2}}$ denote spatial directional derivative operators in two orthogonal directions *φ* and ⊥*φ* aligned with the eigenvectors of the covariance matrix Σ,
• $\partial_{\overset{¯}{t}}=v_{1}\partial_{x_{1}}+v_{2}\partial_{x_{2}}+\partial_{t}$ is a velocity-adapted temporal derivative operator aligned to the direction of the local image velocity $v={\left(v_{1},v_{2}\right)}^{T}$,
• $m_{1}$ and $m_{2}$ denote orders of spatial differentiation,
• *n* denotes the order of temporal differentiation,
• $g(x;\;s\;\Sigma )=\frac{1}{2\pi s\sqrt{\det\Sigma }}e^{-x^{T}\Sigma^{-1}x/2s}$ is an affine Gaussian kernel with its size determined by the spatial scale parameter *s* and its shape determined by the spatial covariance matrix Σ,
• $g(x_{1}-v_{1}t,x_{2}-v_{2}t;\;s\;\Sigma )$ denotes a spatial affine Gaussian kernel that moves with image velocity $v=(v_{1},v_{2})$ in space-time and
• h(t;τ) is a temporal smoothing kernel over time corresponding to a Gaussian kernel $h(t;\;\tau )=g(t;\;\tau )=1/\sqrt{2\pi \tau }\exp(-t^{2}/2\tau )$ in the case of non-causal time or a cascade of first-order integrators or equivalently truncated exponential kernels coupled in cascade $h(t;\;\tau )=h_{composed}(\cdot ;\;\mu )$ according to (27) over a time-causal temporal domain.

This family of spatio-temporal scale-space kernels can be seen as a canonical family of linear receptive fields over a spatio-temporal domain.

**Figure 9:**
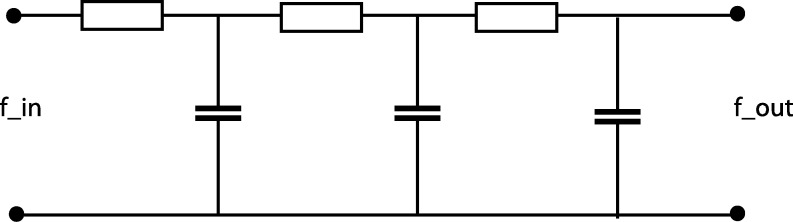
Illustration of the time-causal receptive field model in terms of an electric wiring diagram composed of a set of resistors and capacitors that emulate a series of first-order integrators coupled in cascade. In this model, the time-varying voltage *f*_*in*_ represents the time varying input signal, whereas the time-varying voltage *f*_*out*_ represents the time-varying output signal at a coarser temporal scale. From the theory for temporal scale-space kernels for one-dimensional signals (Lindeberg [21], [56]; Lindeberg and Fagerström [57]), it holds that the corresponding equivalent truncated exponential kernels are the only primitive temporal smoothing kernels that guarantee both temporal causality and non-creation of local extrema (or zero-crossings) with increasing temporal scale.

For the case of a time-causal temporal domain, the result states that truncated exponential kernels of the form

(26) $$h_{exp}(t;\;\mu_{k})=\left\{ \begin{array}{cc} \frac{1}{\mu_{k}}e^{-t/\mu_{k}}\; & t\geq 0\\ 0\; & t>0 \end{array}\right.$$

coupled in cascade constitute the natural temporal smoothing kernels. These do in turn lead to a composed temporal smoothing kernel of the form

(27) $$h_{composed}(\cdot ;\;\mu )={⁎}_{k=1}^{K}h_{exp}(\cdot ;\;\mu_{k})$$

and corresponding to a set of first-order integrators coupled in cascade (see Fig. 9).

Two natural ways of distributing the discrete time constants $\mu_{k}$ over temporal scales are studied in detail in [21], [55] corresponding to either a uniform or a logarithmic distribution in terms of the composed variance

(28) $$\tau_{K}=\sum_{k=1}^{K}\mu_{k}^{2}.$$

Specifically, it is shown in [21, Section 5] that in the case of a logarithmic distribution of the discrete temporal scale levels, it is possible to consider an infinite number of temporal scale levels that cluster infinitely dense near zero temporal scale

(29) $$\ldots \frac{\tau_{0}}{c^{6}},\frac{\tau_{0}}{c^{4}},\frac{\tau_{0}}{c^{2}},\tau_{0},c^{2}\tau_{0},c^{4}\tau_{0},c^{6}\tau_{0},\ldots$$

so that a *scale-covariant time-causal limit kernel* Ψ(t;τ,c) can be defined obeying self-similarity and scale covariance over temporal scales and with a Fourier transform of the form

(30) $$\overset{ˆ}{\Psi }(\omega ;\;\tau ,c)=\prod_{k=1}^{\infty }\frac{1}{1+i\;c^{-k}\sqrt{c^{2}-1}\sqrt{\tau }\;\omega }.$$

**Figure 10:**
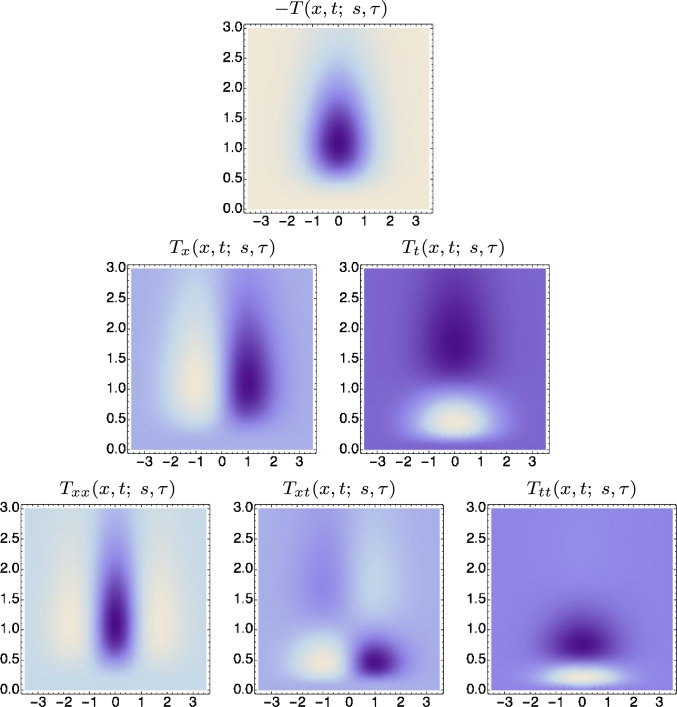
*Illustrations of space-time separable receptive fields*$T_{x^{m}t^{n}}(x,t;\;s,\tau )=\partial_{x^{m}t^{n}}(g(x;\;s)\;h(t;\;\tau ))$ up to order two, formed from by the composition of Gaussian kernels over the spatial domain *x* for spatial scale parameter *s* = 1 and a set of truncated exponential kernels coupled in cascade over the temporal domain *t* according to Equation (27), with a logarithmic distribution of the intermediate temporal scale levels that approximates the time-causal limit kernel in Equation (30) with the following parameters: *τ* = 1, *K* = 7, *c* = 2, *v* = 0. The corresponding family of spatio-temporal receptive fields is closed under spatial scaling transformations as well as under temporal scaling transformations for temporal scaling factors that are integer powers of the distribution parameter *c* of the temporal smoothing kernel. (Horizontal axis: space *x*. Vertical axis: time *t*.)

**Figure 11:**
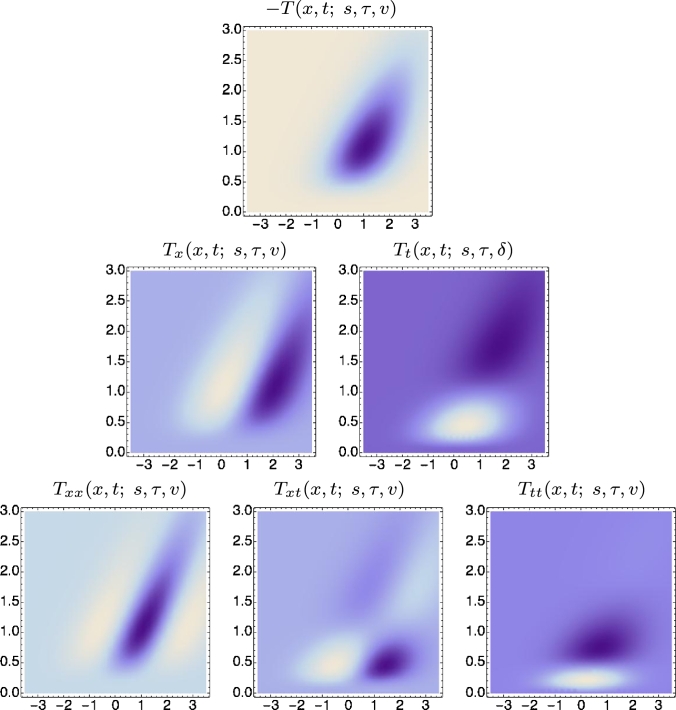
*Illustrations of velocity-adapted spatio-temporal receptive fields*$T_{x^{m}t^{n}}(x,t;\;s,\tau ,v)=\partial_{x^{m}t^{n}}(g(x-vt;\;s)\;h(t;\;\tau ))$ up to order two, formed from the composition of Gaussian kernels over the spatial domain *x* for spatial scale parameter *s* = 1 and a set of truncated exponential kernels coupled in cascade over the temporal domain *t* according to Equation (27), with a logarithmic distribution of the intermediate temporal scale levels that approximates the time-causal limit kernel in Equation (30) with the following parameters: *τ* = 1, *K* = 7, *c* = 2, *v* = 1. In addition to spatial and temporal scaling transformations, the corresponding family of receptive fields is also closed under Galilean transformations. (Horizontal axis: space *x*. Vertical axis: time *t*.)

Fig. 10 and Fig. 11 show spatio-temporal kernels over a 1+1-dimensional spatio-temporal domain using approximations of the time-causal limit kernel for temporal smoothing over the temporal domain and the Gaussian kernel for spatial smoothing over the spatial domain. Fig. 10 shows space-time separable receptive fields corresponding to image velocity v=0, whereas Fig. 11 shows unseparable velocity-adapted receptive fields corresponding to a non-zero image velocity v≠0.

The family of space-time separable receptive fields for zero image velocities is closed under spatial scaling transformations for arbitrary spatial scaling factors as well as for temporal scaling transformations with temporal scaling factors that are integer powers of the distribution parameter *c* of the time-causal limit kernel. The full family of velocity-adapted receptive fields for general non-zero image velocities is additionally closed under Galilean transformations, corresponding to variations in the relative motion between the objects in the world and the observer. Given that the full families of receptive fields are explicitly represented in the vision system, this means that it will be possible to perfectly match receptive field responses computed under the following types of natural image transformations: (i) objects of different size in the image domain as arising from *e.g.* viewing the same object from different distances, (ii) spatio-temporal events that occur with different speed, faster or slower, and (iii) objects and spatio-temporal events that are viewed with different relative motions between the objects/event and the visual observer.

If additionally the spatial smoothing is performed over the full family of spatial covariance matrices Σ, then receptive field responses can also be matched (iv) between different views of the same smooth local surface patch.

### 3.3 Scale normalisation of spatial and spatio-temporal receptive fields

When computing receptive field responses over multiple spatial and temporal scales, there is an issue about how the receptive field responses should be normalized so as to enable appropriate comparisons between receptive field responses at different scales. Issues of scale normalisation of the derivative based receptive fields defined from scale-space operations are treated in [36], [58], [59] regarding spatial receptive fields and in [21], [38], [55] regarding spatio-temporal receptive fields.

*a) Scale-normalized spatial receptive fields.* Let $s_{\varphi }$ and $s_{⊥\varphi }$ denote the eigenvalues of the composed affine covariance matrix *s* Σ in the spatial receptive field model (23) and let $\partial_{\varphi }$ and $\partial_{⊥\varphi }$ denote directional derivative operators along the corresponding eigendirections. Then, the scale-normalized spatial derivative kernel corresponding to the receptive field model (23) is given by

(31) $$T_{\varphi^{m_{1}}⊥\varphi^{m_{2}},norm}(x_{1},x_{2};\;s,\Sigma )=s_{\varphi }^{m_{1}\gamma_{s}/2}\;s_{⊥\varphi }^{m_{2}\gamma_{s}/2}\;\partial_{\varphi }^{m_{1}}\partial_{⊥\varphi }^{m_{2}}\left(g(x_{1},x_{2};\;s\Sigma )\right),$$

where $\gamma_{s}$ denotes the spatial scale normalization parameter of *γ*-normalized derivatives and specifically the choice $\gamma_{s}=1$ leads to maximum scale invariance in the sense that the magnitude response of the spatial receptive field will be preserved under uniform spatial scaling transformations $\left(x_{1}^{\prime },x_{2}^{\prime })=(S_{s}\;x_{1},S_{s}\;x_{2}\right)$, provided that the spatial scale levels are appropriately matched $\left(s_{\varphi }^{\prime },s_{⊥\varphi }^{\prime })=(S_{s}^{2}\;s_{\varphi },S_{s}^{2}\;s_{⊥\varphi }\right)$.

*b) Scale-normalized spatial receptive fields in the case of a non-causal spatio-temporal domain.* For the case of a non-causal spatio-temporal domain, where the temporal smoothing operation in the spatio-temporal receptive field model is performed by a non-causal Gaussian temporal kernel $h(t;\;\tau )=g(t;\;\tau )=1/\sqrt{2\pi \tau }\exp(-t^{2}/2\tau )$, the scale-normalized spatio-temporal derivative kernel corresponding to the spatio-temporal receptive field model (25) is with corresponding notation regarding the spatial domain as in (31) given by

(32) Tφm1⊥φm2t¯n,norm(x1,x2,t;s,τ;v,Σ)=sφm1γs/2s⊥φm2γs/2τnγτ/2=∂φm1∂⊥φm2∂t¯n(g(x1−v1t,x2−v2t;sΣ)h(t;τ)),

where $\gamma_{s}$ and $\gamma_{\tau }$ denote the spatial and temporal scale normalization parameters of *γ*-normalized derivatives and specifically the choice $\gamma_{s}=1$ and $\gamma_{\tau }=1$ leads to maximum scale invariance in the sense that the magnitude response of the spatio-temporal receptive field will be preserved under independent scaling transformations of the spatial and the temporal domains $\left(x_{1}^{\prime },x_{2}^{\prime },t^{\prime })=(S_{s}\;x_{1},S_{s}\;x_{2},S_{\tau }\;t\right)$, provided that both the spatial and temporal scale levels are appropriately matched $\left(s_{\varphi }^{\prime },s_{⊥\varphi }^{\prime },\tau^{\prime })=(S_{s}^{2}\;s_{\varphi },S_{s}^{2}\;s_{⊥\varphi },S_{\tau }^{2}\;\tau \right)$.

*c) Scale-normalized spatial receptive fields in the case of a time-causal spatio-temporal domain.* For the case of a time-causal spatio-temporal domain, where the temporal smoothing operation in the spatio-temporal receptive field model is performed by truncated exponential kernels coupled in cascade $h(t;\;\tau )=h_{composed}(\cdot ;\;\mu )$ (27), the corresponding scale-normalized spatio-temporal derivative kernel corresponding to the spatio-temporal receptive field model (25) is given by

(33) Tφm1⊥φm2t¯n,norm(x1,x2,t;s,τ;v,Σ)=sφm1γs/2s⊥φm2γs/2αn,γτ(τ)=∂φm1∂⊥φm2∂t¯n(g(x1−v1t,x2−v2t;sΣ)h(t;τ)),

where $\gamma_{s}$ and $\gamma_{\tau }$ denote the spatial and temporal scale normalization parameters of *γ*-normalized derivatives and $\alpha_{n,\gamma_{\tau }}(\tau )$ is the temporal scale normalization factor, which for the case of variance-based normalization is given by

(34) $$\alpha_{n,\gamma_{\tau }}(\tau )=\tau^{n\gamma_{\tau }/2}$$

in agreement with (32), while for the case of $L_{p}$-normalization it is given by [21, Equation (76)]

(35) $$\alpha_{n,\gamma_{\tau }}(\tau )=\frac{G_{n,\gamma_{\tau }}}{{\left\|h_{t^{n}}(\cdot ;\;\tau )\right\|}_{p}},$$

with $G_{n,\gamma_{\tau }}$ denoting the $L_{p}$-norm of the *n*:th order scale-normalized derivative of a non-causal Gaussian temporal kernel with scale normalization parameter $\gamma_{\tau }$. In the specific case when the temporal smoothing is performed using the scale-invariant limit kernel (30), the magnitude response will for the maximally scale invariant choice of scale normalization parameters $\gamma_{s}=1$ and $\gamma_{\tau }=1$ be preserved under independent scaling transformations of the spatial and the temporal domains $\left(x_{1}^{\prime },x_{2}^{\prime },t^{\prime })=(S_{s}\;x_{1},S_{s}\;x_{2},S_{\tau }\;t\right)$ for general spatial scaling factors $S_{s}$ and for temporal scaling factors $S_{\tau }=c^{j}$ that are integer powers of the distribution parameter *c* of the scale-invariant limit kernel, provided that both the spatial and temporal scale levels are appropriately matched $\left(s_{\varphi }^{\prime },s_{⊥\varphi }^{\prime },\tau^{\prime })=(S_{s}^{2}\;s_{\varphi },S_{s}^{2}\;s_{⊥\varphi },S_{\tau }^{2}\;\tau \right)$.

### 3.4 Invariance to local multiplicative illumination variations or variations in exposure parameters

The treatment so far has been concerned with modelling receptive fields under natural geometric image transformations, modelled as local scaling transformations, local affine transformations and local Galilean transformations representing the essential dimensions in the variability of a local linearization of the perspective mapping from a local surface patch in the world to the tangent plane of the retina. A complementary issue concerns how to model receptive field responses under variations in the external illumination and under variations in the internal exposure mechanisms of the eye that adapts the diameter of the pupil and the sensitivity of the photoreceptors to the external illumination. In this section, we will present a solution for this problem regarding the subset of intensity transformations that can be modelled as local multiplicative intensity transformations.

To handle image data under illumination variations in a theoretically well-founded manner, it is natural to represent the image data on a logarithmic luminosity scale

(36) $$f(x_{1},x_{2},t)∼\log I(x_{1},x_{2},t).$$

Then, it can be shown that receptive field responses computed from such logarithmic luminosities can be *interpreted physically* as a superposition of relative variations of surface structure and relative variations of illumination variations. To demonstrate why this follows, let us assume: (i) a perspective camera model extended with (ii) a thin circular lens that gathers incoming light from different directions and (iii) a Lambertian illumination model that is complemented with (iv) a spatially varying albedo factor for modelling the light that is reflected from surface patterns in the world. Then, it can be shown [19, Section 2.3] that a spatio-temporal receptive field response

(37) $$L_{\varphi^{m_{1}}⊥\varphi^{m_{2}}{\overset{¯}{t}}^{n}}(\cdot ,\cdot ;\;s,\;\tau )=\partial_{\varphi^{m_{1}}⊥\varphi^{m_{2}}{\overset{¯}{t}}^{n}}\;T_{s,\tau }\;f(\cdot ,\cdot )$$

of the image data *f*, where $T_{s,\tau }$ represents the spatio-temporal smoothing operator (here corresponding to a spatio-temporal smoothing kernel of the form (24)) can be expressed as

(38) Lφm1⊥φm2t¯n(x1,x2,t;s,τ)==∂φm1⊥φm2t¯nTs,τ(log⁡ρ(x1,x2,t)+log⁡i(x1,x2,t)=∂φm1⊥φm2t¯nTs,τ(+log⁡Ccam(f˜(t))+V(x1,x2)),

where

(i) $\rho (x_{1},x_{2},t)$ is a spatially dependent *albedo factor* that reflects *properties of surfaces of objects* in the environment (note that this entity may in general refer to points on different surfaces in the world depending on the viewing direction),
(ii) $i(x_{1},x_{2},t)$ represents a spatially dependent *illumination field* (note that the amount of incoming light may be different for different surfaces world as mapped to corresponding image coordinates $\left(x_{1},x_{2}\right)$ over time *t*),
(iii) $C_{cam}(\overset{˜}{f}(t))=\frac{\pi }{4}\frac{d}{f}$ represents the possibly time-dependent *internal camera parameters* with the ratio $\overset{˜}{f}=f/d$ referred to as the *effective f-number*, where *d* denotes the diameter of the lens and *f* the focal distance, and
(iv) $V(x_{1},x_{2})=-2\log(1+x_{1}^{2}+x_{2}^{2})$ represents a geometric *natural vignetting* effect corresponding to the factor $\log\cos^{4}(\phi )$ for a planar image plane, with *ϕ* denoting the angle between the viewing direction $\left(x_{1},x_{2},f\right)$ and the surface normal (0,0,1) of the image plane. This vignetting term disappears for a spherical camera model.

From the way Equation (38) is structured, we can observe that if we have a non-zero order of spatial differentiation with at least some of $m_{1}>0$ or $m_{2}>0$, then the influence of the internal camera parameters in $C_{cam}(\overset{˜}{f}(t))$ will vanish because of the spatial differentiation with respect to $x_{1}$ or $x_{2}$. In a corresponding manner, the effects of any other multiplicative exposure control mechanism will also vanish. Moreover, for any multiplicative transformation of the illumination field $i^{\prime }(x_{1},x_{2})=C\;i(x_{1},x_{2})$, where *C* is a constant, the logarithmic luminosity will be transformed as $\log i^{\prime }(x_{1},x_{2})=\log C+\log i(x_{1},x_{2})$. This implies that the dependence on *C* will disappear after any spatial or temporal differentiation.

Thus, given that the image measurements are performed on a logarithmic brightness scale, the spatio-temporal receptive field responses will be automatically invariant under local multiplicative illumination variations as well as under local multiplicative variations in the exposure parameters of the retina and the eye.

## 4 Modelling the computational function of biological receptive fields using idealized receptive field profiles

An established methodology to characterize the spatial and temporal response properties of receptive fields in the central visual pathways consists of performing neurophysiological cell recordings of the responses of visual neurons to white noise stimuli. DeAngelis et al. [4], [5] have presented comprehensive surveys of advances made in this way. In these works, the authors emphasize that it is necessary to characterize receptive fields over the *joint* space-time domain, and that it is thus not sufficient to study receptive fields over the spatial domain only. Then, the authors describe basic classes of spatial and spatio-temporal receptive fields in the LGN and the primary visual cortex. Conway and Livingstone [22] and Johnson et al. [23] show results of corresponding investigations concerning spatio-chromatic receptive fields.

In the following, we will outline how the above derived theory for idealized functional models of linear receptive fields can be used for modelling such spatial, spatio-chromatic and spatio-temporal response properties of biological neurons. Indeed, we will show that the derived theory for idealized functional models of linear receptive fields leads to predictions of receptive field profiles that are qualitatively very similar to *all* the linear spatial and spatio-temporal receptive field types presented in (DeAngelis et al. [4], [5]) and also to schematic simplifications of most of the spatio-chromatic receptive fields shown in (Conway and Livingstone [22]) and (Johnson et al. [23]).

### 4.1 Spatial and spatio-temporal receptive fields in the LGN

**Figure 12:**
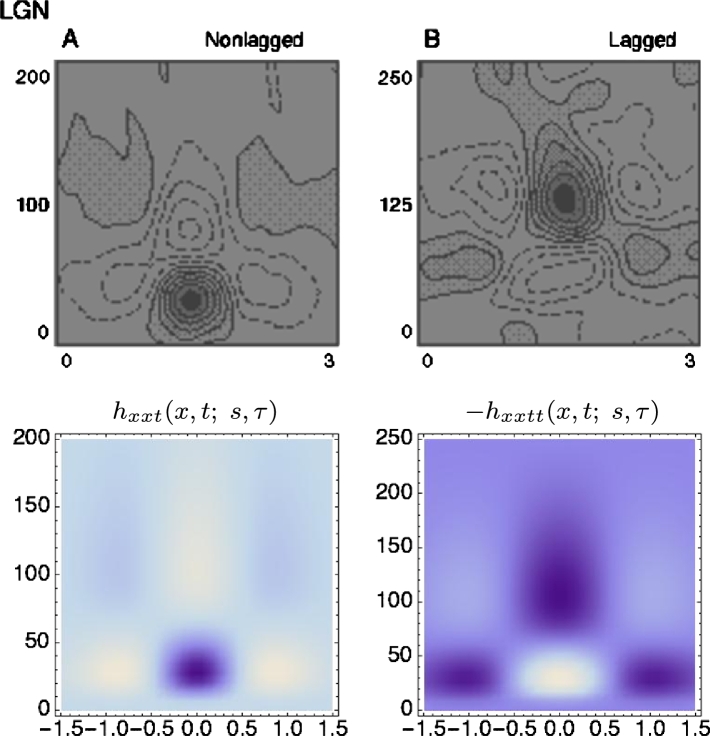
*Spatio-temporal modelling of LGN neurons.* Regarding space-time separable receptive fields in the lateral geniculate nucleus (LGN), there are two main types: For a “non-lagged cell”, the first temporal lobe is strongest, whereas for a “lagged cell”, the second temporal lobe is the strongest one. The top row shows examples of such neurons reported by DeAngelis et al. [4]. In the bottom row, we have modelled these receptive fields by idealized spatio-temporal receptive fields of the form $T(x,t;\;s,\tau )=\partial_{x^{m}}\partial_{t^{n}}(g(;\;s)\;h(t;\;\tau ))$ according to Equation (25), for *m* = 2 corresponding to a Laplacian of Gaussian over the spatial domain, and with the temporal smoothing function *h*(*t*; *τ*) expressed as a cascade of first-order integrators or equivalently truncated exponential kernels of the form (27) and using a logarithmic distribution of the intermediate temporal scale levels. Specifically, in the (left) we model a “non-lagged cell” by first-order temporal derivatives, whereas we model (right) a “lagged cell” using second-order temporal derivatives. Parameter values with $\sigma_{x}=\sqrt{s}$ and $\sigma_{t}=\sqrt{\tau }$: (a) *h*_*xxt*_: *σ*_*x*_ = 0.5 degrees, *σ*_*t*_ = 60 ms, *c* = 2. (b) *h*_*xxtt*_: *σ*_*x*_ = 0.6 degrees, *σ*_*t*_ = 140 ms, *c* = 2. (Horizontal dimension: space *x*. Vertical dimension: time *t*.) (The figures in the top row are reprinted with permission.)

Neurophysiological studies by DeAngelis et al. [4], [5] and others report that most neurons in the lateral geniculate nucleus (LGN), have receptive fields (i) with approximately circular center-surround organization over image space (ii) and that they are separable over space-time. Furthermore, they are two main types of temporal responses: (i) for a “non-lagged cell” the first temporal lobe is the strongest one (Fig. 12(left)), whereas (ii) for “lagged cell” the second temporal lobe is strongest (Fig. 12(right)), see also Ghodrati et al. [53] for a more extensive overview of properties of LGN neurons

When using a time-causal temporal smoothing kernel, the first peak of a first-order temporal derivative will be strongest, whereas the second peak of a second-order temporal derivative will be strongest (see [21, Fig. 2]). Thus, according to this theory, non-lagged LGN cells can be seen as corresponding to first-order time-causal temporal derivatives, whereas lagged LGN cells can be seen as corresponding to second-order time-causal temporal derivatives.

Comparing to the proposed framework for idealized receptive fields, the spatial response of such a neuron is highly similar to a Laplacian of a Gaussian, which leads to a composed idealized receptive field model of the form [19, Equation (108)]

(39) $$h_{LGN}(x,y,t;\;s,\tau )=\pm (\partial_{xx}+\partial_{yy})\;g(x,y;\;s)\;\partial_{t^{n}}\;h(t;\;\tau ).$$

**Figure 13:**
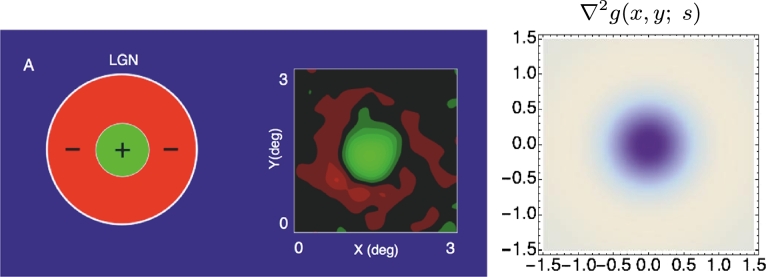
*Spatial modelling of LGN neurons.* (left) DeAngelis et al. [4] report that LGN neurons have approximately circular center-surround responses over the spatial domain. (right) In terms of our idealized receptive field models, such a spatial dependency can be modelled by the Laplacian of the Gaussian $\nabla^{2}g(x,y;\;s)=(x^{2}+y^{2}-2s)/(2\pi s^{3})\exp(-(x^{2}+y^{2})/2s)$, here with $\sigma_{s}=\sqrt{s}=0.6$ in units of degrees of visual angle. (Left and middle figures reprinted with permission.)

In Fig. 13, we show the result of modelling the spatial component of a receptive field in the LGN with a Laplacian of the Gaussian. Such a Laplacian of the Gaussian is also applicable for spatial modelling of on-center/off-surround and off-center/on-surround receptive fields in the retina. In Fig. 12, we show results of joint spatio-temporal modelling of space-time separable receptive fields in the LGN, with the temporal smoothing over the temporal domain expressed as a cascade of truncated exponential kernels of the form (27) and complemented by first- or second-order derivatives.

In previous work by (Rodieck [29]), differences of Gaussians have been shown to constitute a very good approximation of the spatial component of receptive fields in the retina and the LGN. The Laplacian of Gaussian model $\left(\partial_{xx}+\partial_{yy})\;g(x,y;\;s\right)$ over the spatial domain is closely related to such differences of Gaussians. This relationship can be shown from fact that the rotationally symmetric Gaussian satisfies the isotropic diffusion equation [37]:

(40) $$\frac{1}{2}\nabla^{2}L(x,y;\;s)=\partial_{s}L(x,y;\;s)\approx \frac{L(x,y;\;s+\Delta s)-L(x,y;\;s)}{\Delta s}=\frac{DOG(x,y;\;s,\Delta s)}{\Delta s}.$$

This relationship means that differences of Gaussians approximate derivatives over scale, which in turn correspond to Laplacian responses. Conceptually, this implies very good agreement with the spatial component of the LGN model (39) based on Laplacians of Gaussians. In more recent work, Bonin et al. [60] has also found that LGN responses in cats can be well modelled by differences of Gaussians in combination with temporal smoothing, also complemented by a non-linear contrast gain control mechanism (which we do not model specifically here, although the logarithmic brightness scale considered in this treatment will handle variabilities in illumination that could also be handled by non-linear gain control).

### 4.2 Double-opponent spatio-chromatic receptive fields in the LGN

**Figure 14:**
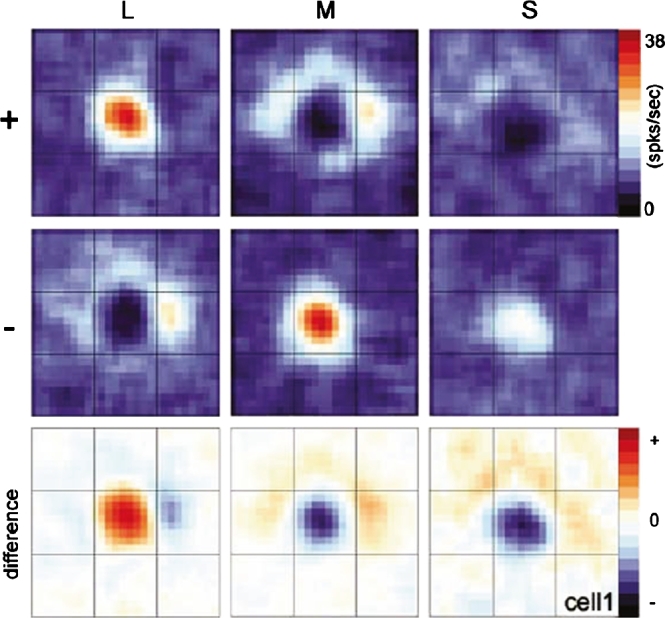
*Receptive field responses of a spatio-chromatic double-opponent neuron* according to Conway and Livingstone [22, Fig. 2, Page 10831]. Here, the colour channels L, M and S basically correspond to red, green and blue colour channels, respectively, from which corresponding red/green and yellow/blue colour-opponent channels can be computed from the difference between L to M and the difference between L+M to S, respectively.

Conway and Livingstone [22] have presented a study of spatio-chromatic response properties of V1 neurons in the alert Macaque monkey. They report the finding of *double-opponent cells*, that simultaneously compute both spatial and chromatic opponency. These cells have receptive fields with approximately circular red/green and yellow/blue colour-opponent response properties, see Fig. 14, and which are claimed to constitute the first layer of spatially opponent colour computations.

**Figure 15:**
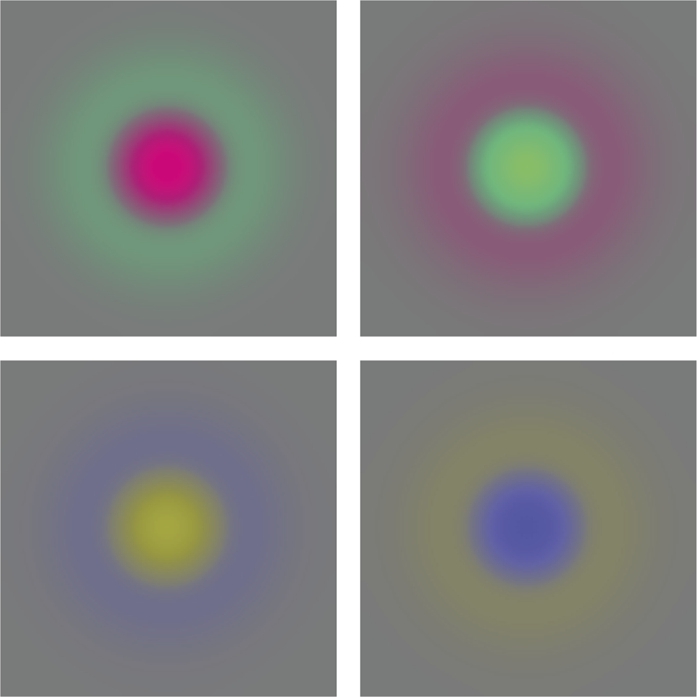
*Modelling of double-opponent neurons using idealized spatio-chromatic receptive fields over the spatial domain.* Here, we have applied the spatial Laplacian operator to positive and negative red/green and yellow/blue colour opponent channels, respectively. These receptive fields can be seen as idealized models of the spatial component of double-opponent spatio-chromatic receptive fields in the LGN.

If we in analogy with the previous modelling of rotationally symmetric on-center/off-surround and off-center/on-surround receptive fields in the LGN by Laplacian of Gaussians (39), apply the Laplacian of the Gaussian operator to red/green and yellow/blue colour-opponent channels,

(41) $$\left(\begin{array}{c} f\\ u\\ v \end{array}\right)=\left(\begin{array}{ccc} \frac{1}{3}\; & \frac{1}{3}\; & \frac{1}{3}\\ \frac{1}{2}\; & -\frac{1}{2}\; & 0\\ \frac{1}{2}\; & \frac{1}{2}\; & -1 \end{array}\right)\left(\begin{array}{c} R\\ G\\ B \end{array}\right),$$

respectively, we get equivalent spatio-chromatic receptive fields that correspond to red-center/green-surround, green-center/red-surround, yellow-center/blue-surround or blue-center/yellow-surround, respectively, see Fig. 15. This corresponds to applying the following spatio-chromatic receptive field model to the RGB channels

(42) $$h_{double-opponent}(x,y;\;s)=\pm (\partial_{xx}+\partial_{yy})\;g(x,y;\;s)\left(\begin{array}{ccc} \frac{1}{2}\; & -\frac{1}{2}\; & 0\\ \frac{1}{2}\; & \frac{1}{2}\; & -1 \end{array}\right),$$

and which constitutes an idealized model for the spatio-chromatic response properties of double-opponent cells.

### 4.3 Spatial, spatio-chromatic and spatio-temporal receptive fields in V1

In their study of neurons in the primary visual cortex (V1), DeAngelis et al. [4], [5] report that the receptive fields of V1 neurons, in general, have different response properties compared to LGN neurons in the following ways: (i) they are oriented in the spatial domain and (ii) they are sensitive to specific ranges of stimulus velocities. According to the pioneering work by Hubel and Wiesel [1], [2], [3]), simple cells are additionally characterized by the following properties: (iii) they have precisely localized “on” and “off” subregions, (iv) spatial summation takes place over each subregion, (v) there is spatial antagonism between on- and off-subregions, and (vi) visual responses to stationary or moving spots can be predicted from the spatial subregions.

**Figure 16:**
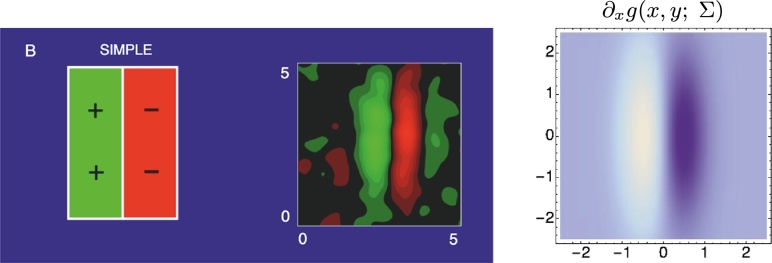
Computational modelling of a receptive field profile over the spatial domain in the primary visual cortex (V1) as reported by DeAngelis et al. [4], [5] using affine Gaussian derivatives: (middle) Receptive field profile of a simple cell over image intensities as reconstructed from cell recordings, with positive weights represented as green and negative weights by red. (left) Stylized simplification of the receptive field shape. (right) Idealized model of the receptive field from a first-order directional derivative of an affine Gaussian kernel ∂_*x*_*g*(*x*,*y*; Σ)=∂_*x*_*g*(*x*,*y*; *λ*_*x*_,*λ*_*y*_) according to (21), here with $\sigma_{x}=\sqrt{\lambda_{x}}=0.5$ and $\sigma_{y}=\sqrt{\lambda_{y}}=1.5$ in units of degrees of visual angle, and with positive weights with respect to image intensities represented by white and negative values by violet. (Left and middle figures reprinted with permission.)

**Figure 17:**
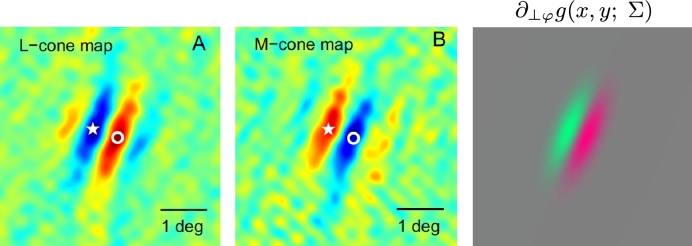
Modelling of double-opponent simple cells in the primary visual cortex (V1) in terms of affine Gaussian derivatives over colour-opponent channels, based on neurophysiological cell recordings by Johnson et al. [23]: (left) Responses to L-cones corresponding to long wavelength red cones, with positive weights represented by red and negative weights by blue. (middle) Responses to M-cones corresponding to medium wavelength green cones, with positive weights represented by red and negative weights by blue. (right) Idealized model of the receptive field from a first-order directional derivative of an affine Gaussian kernel ∂_⊥*φ*_*g*(*x*,*y*; Σ) according to (21) over a red-green colour-opponent channel for $\sigma_{1}=\sqrt{\lambda_{1}}=0.6$ and $\sigma_{2}=\sqrt{\lambda_{2}}=0.2$ in units of degrees of visual angle, *α* = 67 degrees and with positive weights for the red-green colour-opponent channel represented by red and negative values by green. (Left and middle figures: Copyright 2008 of Society for Neuroscience with permission.)

Fig. 16 shows an example of the spatial dependency of a simple cell, that can be well modelled by a first-order affine Gaussian derivative over image intensities. Fig. 17 shows corresponding results for a colour-opponent receptive field of a simple cell in V1, that can be modelled as a first-order affine Gaussian spatio-chromatic derivative over an R-G colour-opponent channel.

Biological support for using multiple affine receptive fields, over an expansion of the shapes of the affine covariance matrices Σ, can be obtained from neurophysiological measurements by Goris et al. [50], who show that there is a large variability in the orientation selectivity of simple and complex cells (see Fig. 19). With regard to the presented theoretical model for simple cells in Equation (23), possibly extended with a colour-opponent representation (41) for spatio-chromatic receptive fields, this means that we could think of all affine receptive fields, with their directional derivatives in preferred directions aligned to the eigendirections of the covariance matrix Σ, as being present at any position in the image domain (see Fig. 8). Such a variability makes it possible to perfectly match the first-order variability of receptive field responses under variations of the slant and tilt directions of a smooth surface patch.

**Figure 18:**
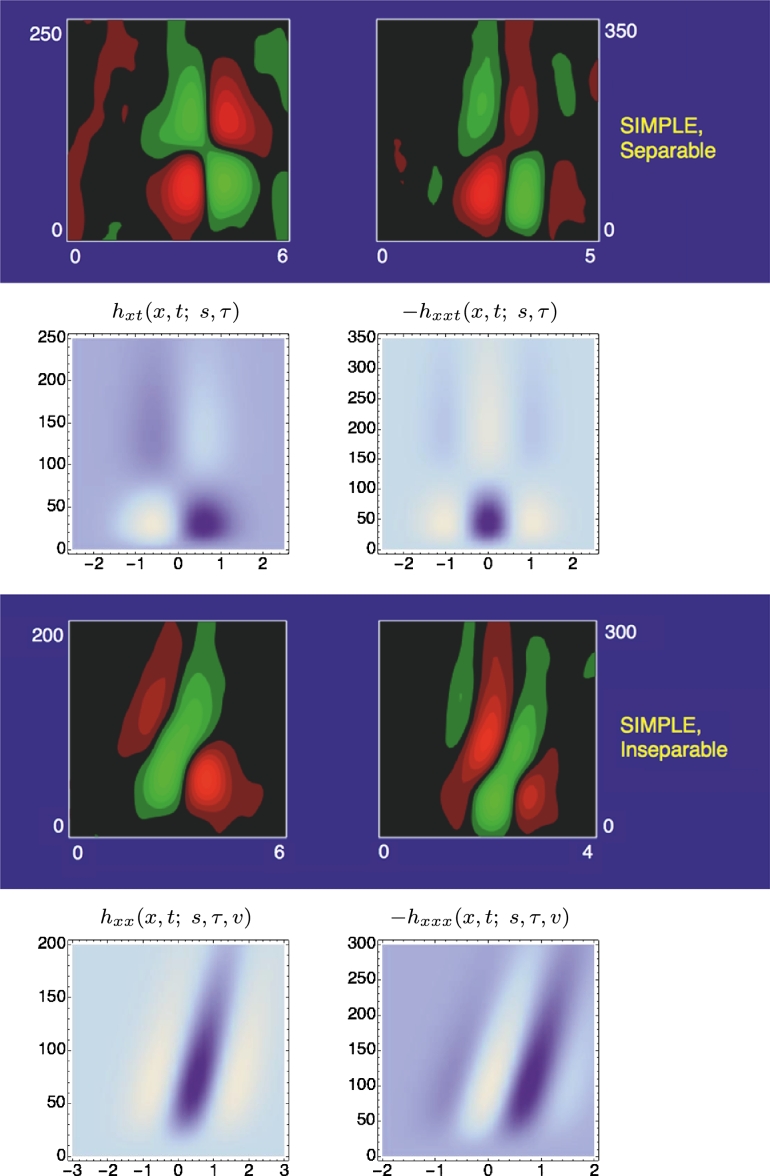
Modelling of space-time separable and inseparable simple cells in the primary visual cortex (V1) based on neural cell recordings reported by DeAngelis et al. [4]. The idealized spatio-temporal receptive fields are of the form $T(x,t;\;s,\tau ,v)=\partial_{x^{m}}\partial_{t^{n}}(g(x-vt;\;s)\;h(t;\;\tau ))$ according to Equation (25), where *v* = 0 corresponds to space-time separable receptive fields and *v* ≠ 0 to inseparable receptive fields. The temporal smoothing function *h*(*t*; *τ*) is modelled as a set of first-order integrators/truncated exponential kernels of the form (27) coupled in cascade and using a logarithmic distribution of the intermediate temporal scale levels. (upper left) Separable receptive fields corresponding to first-order derivatives with respect to space and time. (upper right) Separable receptive fields corresponding to second-order derivatives with respect to space and first-order derivatives with respect to time. (lower left) Inseparable velocity-adapted receptive fields corresponding to second-order derivatives over space. (lower right) Inseparable velocity-adapted receptive fields corresponding to third-order derivatives over space. Parameter values with $\sigma_{x}=\sqrt{s}$ and $\sigma_{t}=\sqrt{\tau }$: (a) *h*_*xt*_: *σ*_*x*_ = 0.6 degrees, *σ*_*t*_ = 80 ms, *c* = 2. (b) *h*_*xxt*_: *σ*_*x*_ = 0.6 degrees, *σ*_*t*_ = 120 ms, *c* = 2. (c) *h*_*xx*_: *σ*_*x*_ = 0.7 degrees, *σ*_*t*_ = 70 ms, *v* = 0.007 degrees/ms, *c* = 2. (d) *h*_*xxx*_: *σ*_*x*_ = 0.5 degrees, *σ*_*t*_ = 100 ms, *v* = 0.004 degrees/ms, *c* = 2. (Horizontal axis: Space *x* in degrees of visual angle. Vertical axis: Time *t* in ms.) (The figures in the top and third rows reprinted with permission.)

In Fig. 18, we show spatio-temporal dependencies of a set of separable and inseparable simple cells in V1 that can be modelled using the general idealized model of spatio-temporal receptive fields in Equation (25), based on Gaussian derivatives over image space and temporal derivatives of a set of truncated exponential kernels coupled in cascade (27). The results in the upper part show space-time separable spatio-temporal receptive fields corresponding to zero image velocity v=0, and corresponding to either first- or second-order spatial derivatives over image space in combination with first-order temporal derivatives over time. The results in the lower part show inseparable spatio-temporal receptive fields corresponding to non-zero image velocities and based on either second- or third-order spatial derivatives over image space.

**Figure 19:**
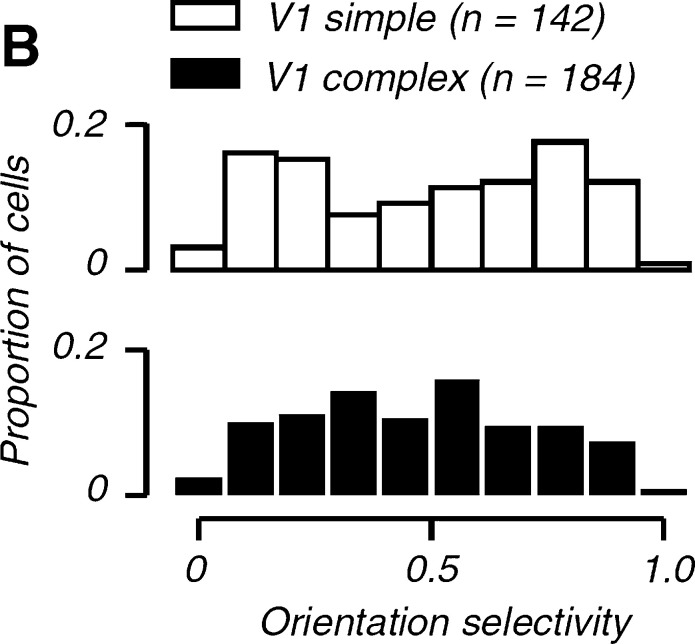
Measurements of the orientation selectivity of simple cells and complex cells in the primary visual cortex of the Macaque monkey as reported by Goris et al. [50]. Interpreted with regard to the affine Gaussian derivative model for the receptive fields of simple cells (23), this large variability in orientation selectivity implies that we should consider covariance matrices Σ for a large range of eccentricities, as can be quantified by ratio between their eigenvalues *λ*_1_ and *λ*_2_. (The orientation selectivity of an affine Gaussian derivative kernel increases with the eccentricity.)

To conclude, from these figures we can see that the qualitative shape of biological receptive fields, as recorded by neurophysiological measurements, can be quite well modelled by the proposed idealized receptive field models that result from the presented normative theory of visual receptive fields.

## 5 Relations to previous work

In earlier work, Young [30] has also proposed to model spatial visual receptive fields by Gaussian derivatives and shown that visual receptive fields in cats and monkeys can be well modelled by Gaussian derivatives up to order four. Young et al. [31], [32] have also proposed to model spatio-temporal receptive fields by Gaussian derivatives over the spatio-temporal domain. This corresponds to the non-causal purely Gaussian spatio-temporal concept presented in this article, as well as in our closely related earlier work [61], [62]. Young does, however, use a different type of parameterization.

The normative theory for visual receptive fields presented in [13], [19], [20], [21] and here does first of all provide additional theoretical foundation for Young's spatial modelling work based on Koenderink and van Doorn's theory [8], [9]. It does additionally extend that model from regular (isotropic) Gaussian derivatives to affine Gaussian derivatives, and does also provide a conceptual extension to a time-causal spatio-temporal domain that takes into explicit account the fact that the future cannot be accessed. Furthermore, our model provides a better parameterization of the spatio-temporal receptive field model over a non-causal spatio-temporal domain based on the Gaussian spatio-temporal scale-space concept.

This model, or earlier versions of it, has in turn been exploited for modelling of biological receptive fields by Lowe [63], May and Georgeson [64], Hesse and Georgeson [65], Georgeson et al. [66], Wallis and Georgeson [67], Hansen and Neumann [68], Wang and Spratling [69], Mahmoodi [70], [71] and Pei et al. [72].

### 5.1 Relations to modelling by Gabor functions

Motivated by the property of Gabor functions [73]

(43) $$G(x;\;s,\omega )=e^{-i\omega x}\;g(x;\;s),$$

that they minimize the uncertainty relation, Marčelja [24], Jones and Palmer [25], [26], Ringach [27], [28] and others have proposed to use Gabor functions to model spatial dependencies of visual receptive fields. There are, however, reasons to question this motivation, both on theoretical and empirical grounds: Following the arguments by Stork and Wilson [74]: (i) only the full complex-valued Gabor functions (treated as pairs) minimize the uncertainty relation, the single real or imaginary components do not, (ii) the real-valued functions that minimize the uncertainty relation are Gaussian kernels and Gaussian derivatives, not Gabor functions, (iii) quantitative comparisons between Gabor functions and other functions to physiological and psychophysical data have demonstrated that other functions, such as Gaussian derivatives, may enable better fits between the model and the data than for Gabor functions.

There are conceptual similarities between Gabor functions and Gaussian derivatives in terms of the ripples they have. For Gabor functions, the ripples are given by the zero-crossings of complex sine waves. For Gaussian derivatives, the ripples are given by the zero-crossings of Hermite functions of different orders. To specify a Gabor function, does, however, require two parameters; a scale parameter representing the spatial extent and a frequency. To specify a Gaussian derivative requires a scale parameter and the order of (spatial or temporal) differentiation, The Gaussian derivative model has the theoretically attractive properties that the receptive fields satisfy the diffusion equation and that derivatives of different orders can be mutually related by derivative operators, and can be computed from local nearest-neighbour operations over image space, implying that they can be implemented in biological wetware by connections between neighbouring computational units (neurons).

Regarding invariance properties to natural image transformations, it holds that the family of affine Gaussian kernels is closed under the full group affine image deformations. The family of Gabor functions based on multiplications of rotationally symmetric Gaussians with sine and cosine waves is not closed under general affine image deformations. This implies that we cannot compute truly affine invariant image representations from such families of traditional Gabor functions. If we have a pair of images that are related by a non-uniform scaling transformation, then the lack of affine covariance means that there will be systematic errors if we attempt to match image representations that are computed from such Gabor functions. If we compute receptive field responses based on directional derivatives of affine Gaussian kernels, it will on the other hand be possible to compute fully affine invariant features [20], in turn providing better internal consistency between receptive field responses computed from different views of objects in the world.

Concerning invariance to multiplicative illumination variations, it holds that the integral of the even cosine component of a Gabor function will, in general, not be equal to zero. This implies that the illumination invariant properties under local multiplicative illumination transformations or multiplicative exposure control mechanisms outlined in Section 3.4 will not hold for receptive field responses that are computed from such Gabor functions.

In these respects, the proposed Gaussian derivative model is conceptually simpler, the image measurements can be theoretically modelled using tools in differential geometry, it can be derived by necessity from symmetry principles in an axiomatic manner, its receptive field responses can be computed from local connections, and it enables provable invariance properties under local linearized image deformations (affine transformations) as well as to local multiplicative illumination variations and multiplicative exposure control mechanisms.

### 5.2 Relations to approaches for learning receptive fields from natural image statistics

**Figure 20:**
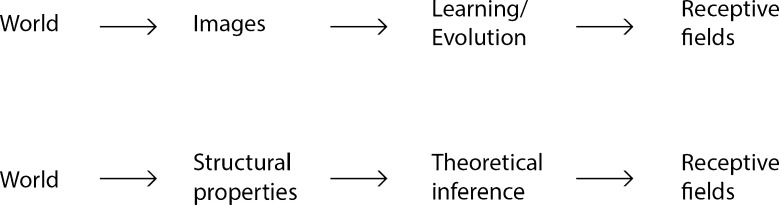
Two structurally different ways of deriving receptive field shapes for a vision system intended to infer properties of the world by either biological or artificial visual perception. (top row) A traditional model for learning receptive fields shapes consists of collecting real-world image data from the environment, and then applying learning algorithms possibly in combination with evolution over multiple generations of the organism that the vision system is a part of. (bottom row) With the normative theory for receptive fields presented in this paper, a short-cut is made in the sense that the derivation of receptive field shapes starts from structural properties of the world (corresponding to symmetry properties in theoretical physics) from which receptive field shapes are constrained by theoretical mathematical inference.

A more data-driven approach to defining receptive field models that has been explored in the field consists of learning them from the statistics of natural image data (Field [75]; van der Schaaf and van Hateren [76]; Olshausen and Field [77]; Rao and Ballard [78]; Simoncelli and Olshausen [79]; Geisler [80]; Hyvärinen et al. [81]; Lörincz [82]). This approach also leads to visual receptive fields with similar shapes as those found in biological vision. The presented theory of visual receptive fields can in this context be seen as constituting a meta theory that describes the fundamental physical constraints under which different learning based method will operate. The physical structure of the world will determine what types of natural images can be generated. Field [75] and Doi and Lewicki [83] have described how “natural images are not random, instead they exhibit statistical regularities”. If a learning system is assumed to be optimally adapted, we could expect that the receptive field shapes it learns should agree with the theoretical predictions, provided that the data sets for learning are sufficiently large and sufficiently representative with regard to the properties of natural image data (see Fig. 20).

The theory proposed in this paper can thus be interpreted as a theory at a higher level of abstraction, formulated based on basic principles that reflect properties of the environment, which in turn determine properties of the natural image data, and with no need for explicit statistical modelling of the image data. Specifically, the presented theory explains why statistical approaches for learning receptive fields can be expected to lead to qualitatively similar models for receptive fields as the idealized functional models of receptive fields.

From the observation that the receptive field profiles in the retina, the LGN and the primary visual cortex of higher mammals are very close to *ideal*, in the sense that the biologically measured receptive fields are very similar to the predictions from the idealized theory, we can regard biological vision as having adapted very well to the transformation properties of the outside world, specifically the transformations corresponding to the mapping of the three-dimensional world to two-dimensional images. This property may be regarded as highly desirable for a biological organism, if there is or has been sufficient evolutionary pressure on its vision system.

### 5.3 Logarithmic brightness scale

Concerning the concept of a *logarithmic brightness scale*, already the Greek astronomer Hipparchus implicitly made use of this notion, when he defined a subjective scale for the brightness of stars. In his brightness scale, divided into six levels, the brightest stars were referred to as of the first magnitude, whereas the faintest stars, near to what can be perceived by a human observer without additional lenses, were said to be of the sixth magnitude. Later, when it became possible to accurately measure the physical intensities of stars, it was noted that Hipparchus subjective scale corresponded to logarithmic intensity values. Today, in astronomy, the *apparent brightness* of stars is still quantified in terms of logarithmic intensities, although over a much wider range of brightness. The retinex theory of early vision (Land [84], [85]) does also make use of a logarithmic brightness scale.

A logarithmic relationship between the perceived intensity and the physical magnitude of stimuli does more generally occur in the *Weber-Fechner law* in psychophysics. Consider a background intensity *I* that is subject to an increment threshold Δ*I* corresponding to a just noticeable difference. Then, the Weber-Fechner law states that the Weber ratio intensity *I*

(44) $$\frac{\Delta I}{I}=k,$$

is constant over large ranges of magnitude variations [86, Pages 671–672]. The theoretical analysis in Section 3.4, regarding invariance properties of a logarithmic brightness scale under multiplicative intensity transformations and multiplicative exposure control mechanisms, is in excellent agreement with these psychophysical findings. If one considers an adaptive image exposure mechanism in the retina that adapts the diameter of the pupil and the sensitivity of the photopigments, such that relative range variability in the signal divided by the mean illumination is held constant (44) (see *e.g.* Peli [87]), then such an adaptation mechanism can be seen as implementing an approximation of the derivative of a logarithmic transformation

(45) $$d(\log z)=\frac{dz}{z}.$$

This result is also closely related to information theoretic arguments by (Jaynes [88]) to use log⁡z as a default parameterization of a strictly positive entity, in the absence of further information. Then, the ratio dz/z becomes a dimensionless integration measure.

The physical model in Section 3.4 provides a formal justification for transforming brightness values in a logarithmic way in connection to receptive field measurements, and how such a transformation relates to inherent physical properties of object surfaces in the environment.

## 6 Summary

From neurophysiological cell recordings we know that mammalian vision has developed receptive fields with characteristic properties: The first layers of visual receptive fields are tuned to different sizes and orientations over the spatial domain, and to different image velocities over joint space-time. In this article, we have presented an overview of a normative theory that shows how it is possible to derive such receptive field profiles *by necessity*, starting from a set of structural requirements of an *idealized vision system*, and whose functionality is determined by set of mathematical and physical assumptions (see Fig. 20).

These structural requirements reflect *structural properties of the world* for the receptive fields to be compatible with natural image transformations including: (i) variations in the sizes of objects in the world, (ii) variations in the viewing distance, (iii) variations in the viewing direction, (iv) variations in the relative motion between objects in the world and the observer, (v) variations in the speed by which temporal events occur and (vi) local multiplicative illumination variations or multiplicative exposure control mechanisms.

We argue that it is natural for vision system, that is to *interact with the world* in a successful manner, to adapt to these structural requirements. If there is sufficient evolutionary pressure on an organism, in competition between different individuals of the same species or between individuals of different species, adaptation to the principles that determine structural properties of the environment may constitute an *evolutionary advantage*.

The proposed *normative theory* provides a way to derive *functional models of linear receptive fields* from first principles, leading to receptive field shapes in terms of affine Gaussian derivatives and closely related operators. Specifically, the presented theory can *explain* the different shapes of receptive field profiles that are found in biological vision from a requirement that the visual system should be able to compute covariant receptive field responses under the natural types of image transformations that occur in the environment, to enable the computation of invariant representations for perception at higher levels in the visual hierarchy [20] (see Appendix I in the supplement for a description about how covariant receptive fields at lower layers in the visual hierarchy enable invariances to geometric image transformations at higher levels in the visual hierarchy).

Such a view, that V1 performs an expansion of image data over the parameters of natural image transformations, is consistent with the substantial expansion of measurement data that is performed from the LGN^8^ with about 1 M neurons and 1 M output channels to V1 with 190 M neurons and 37 M output channels [89, Fig. 3].

We have shown that the predictions from the presented theory are in good qualitative agreement with receptive fields found by neurophysiological cell recordings in mammalian vision. Specifically, we have presented idealized functional models (i) for space-time separable receptive fields in the retina and the LGN and (ii) for both space-time separable and non-separable simple cells in the primary visual cortex (V1).

The qualitatively very good agreement between the predicted receptive field profiles from the normative axiomatic theory with the receptive field profiles found by neurophysiological measurements indicates that the earliest receptive fields in higher mammal vision can be interpreted as having reached a state that can be seen as very close to *ideal* in view of the stated structural requirements/symmetry properties. From this viewpoint, mammalian vision can be interpreted as having adapted very well to the transformation properties of the outside world and to the transformations that occur when a three-dimensional world is projected onto a two-dimensional image domain.

In relation to other approaches of learning receptive field profiles from natural image statistics, the presented theory determines receptive field shapes without any need for training data. The presented theoretical approach also adds explanatory value in terms of underlying covariance and invariance properties, in the sense that requiring the first layers of receptive fields to be provably covariant under scaling transformations, rotations, perspective transformations and Galilean transformations makes it possible to define invariant properties with respect to these essential transformation groups at higher levels in the visual hierarchy. If the underlying first layers of visual receptive fields would not obey such covariance properties, then there would be a systematic bias in the visual operations, corresponding to the amount of mismatch between the backprojected receptive fields.

Corresponding types of arguments applied to the area of hearing, lead to the formulation of a normative theory of auditory receptive fields (Lindeberg and Friberg [90], [91]).

## Declarations

### Author contribution statement

Tony Lindeberg: Conceived and designed the experiments; Performed the experiments; Analyzed and interpreted the data; Contributed reagents, materials, analysis tools or data; Wrote the paper.

### Funding statement

This work was supported by the 10.13039/501100004359Swedish Research Council (Grant Numbers 2014-4083, 2018-03586).

### Data availability statement

Data included in article/supplementary material/referenced in article.

### Declaration of interests statement

The authors declare no conflict of interest.

### Additional information

Supplementary content related to this article has been published online at https://doi.org/10.1016/j.heliyon.2021.e05897.

No additional information is available for this paper.

## Appendix Supplementary material

heliyon-suppl.pdf This supplement contains theoretical material for the paper Lindeberg (2021) “Normative theory of visual receptive fields” published in Heliyon. heliyon-suppl.pdf

## Footnotes

1 Specifically, both the spatial and temporal smoothing and the computation of spatial and temporal derivatives, that constitute the computational primitives in the receptive field models that we will arrive at, can be performed by local connections between neighbouring neurons in our time-causal model.

2 While it is known that many receptive fields may have both linear and non-linear components, we do here, as a conceptual simplification, focus on the regime where receptive fields in the LGN and the primary visual cortex can be well approximated by a linear model. Note, however, that this assumption about linearity of the first layers of visual receptive fields does not exclude the possibility of defining later stage non-linear receptive fields that operate on the output from the linear receptive fields, such as the computations performed by complex cells in the primary visual cortex [1], [2], [39], [40], [41], [42], [43], [44], [45], [46], [47], [48], [49], [50], [51]. Specifically, in [51, Section 5] a functional energy model of complex cells is proposed, based on simple cell responses modelled by affine Gaussian derivatives as described in this work. Neither does the assumption of linearity exclude the possibility of transforming the raw image intensities by a pointwise non-linear mapping function prior to the application of linear receptive fields over local neighbourhoods. In Section 3.4, it will be specifically shown that a pointwise logarithmic transformation of the image intensities prior to the application of linear receptive fields has theoretical advantages of enabling invariance properties of derivative-based receptive field responses under local multiplicative illumination transformations.

3 Concerning the use of a linear model for the receptive fields in the retina used in this paper, it should be noted that there are also more sophisticated non-linear computations known to be performed in the retina [52]. Light adaptation and gain control in the retina, we model by an initial logarithmic transformation prior to the receptive fields, which has the desirable property of leading to invariant receptive field responses under multiplicative illumination transformations, as will be described in Section 3.4. Direction sensitive cells that respond to different directions of motion, to handle objects that move rapidly over the visual field and to decrease temporal delays in a time-causal context by the ability of performing motion extrapolation to perform temporal predictions, we capture some of the effects of by studying the influence of Galilean transformations, Probably, the function of such non-linear mechanisms could also be constrained from structural properties of the world, in a similar way as we here constrain the shapes of visual receptive fields from theoretical assumptions. The use of a pure linear model in this treatment, should in this context be seen as a conceptual simplification and a way to simplify the theoretical analysis.

4 Concerning the use of a pure feed-forward model of the receptive fields used in this paper, it should be noted that there is a also a large number of top-down connections to the LGN from higher visual areas [53], which we do not explicitly model in this treatment.

5 The formal derivation of this result concerning spatial receptive fields is based on two main conceptual steps: In a first stage, we establish that from the assumptions of linearity and translational covariance in combination with a semi-group structure over a continuous scale parameter and certain regularity assumptions in terms of Sobolev norms, the internal scale-space representations according to (1) must obey an evolution equation over scale of the form $\partial_{s}L=AL$ for some linear and translation covariant operator A, see [13, Lemma 2]. In a second stage, we show that by adding the requirement of non-enhancement of local extrema, the operator A may only correspond to second- and first-order derivatives at the central point, corresponding to a diffusion equation of the form (18), see [13, Theorem 5].

6 For the theoretical analysis in this treatment, we consider the affine diffusion equation as defined over the entire infinite image plane, $x={\left(x_{1},x_{2}\right)}^{T}\in R^{2}$, and normally with initial equation $L(x_{1},x_{2};s)=f(x_{1},x_{2})$ for s=0. When using this equation to model the effect of visual operations for simulation experiments on finite images, we solve this equation with adiabatic boundary conditions, corresponding to no heat flow across the image boundaries, and equivalent to reflections of the image data at the image boundaries, when implementing the spatial smoothing operation in terms of explicit Gaussian filtering. This turns out to be a good approximation for moderate fields of view. An alternative approach for handling large fields of view is by reformulating this equation on a sphere, corresponding to computations on the viewsphere.

8 In the retina, there are about 100 M photoreceptors with about 1 M output channels to the LGN.

## Footnotes

7 Here, we parameterize the spatial scale parameter in units of *s*, where *s* after the mathematical derivations turns out to have dimension ${\left[\text{length}\right]}^{2}$. With this parameterization, the scale values become additive under the convolution operation (5), because of the semi-group property in Equation (6). The scale values in units of *s* also become additive under evolution according to the diffusion equation (18). If two Gaussian convolutions with scale parameters $s_{1}$ and $s_{2}$ are performed after each other, the composed smoothing effect is $s_{3}=s_{1}+s_{2}$. If the scale parameters would instead have been measured in units of $\sigma =\sqrt{s}$, with *σ* having dimension [length], then the corresponding composed smoothing effect would instead be written $\sigma_{3}=\sqrt{\sigma_{1}^{2}+\sigma_{2}^{2}}$.
