# Supplementary material for: Normative theory of visual receptive fields
Source: Heliyon. 2021 Jan 21;7(1):e05897. doi: 10.1016/j.heliyon.2021.e05897 (PMC7820928; doi:10.1016/j.heliyon.2021.e05897)
Supplement: heliyon-suppl.pdf — This supplement contains theoretical material for the paper Lindeberg (2021) “Normative theory of visual receptive fields” published in Heliyon. [file mmc1.pdf]

Supplement to "Normative theory of visual receptive fields": Appendix

Tony Lindeberg
Computational Brain Science Lab, Division of Computational Science and Technology,
KTH Royal Institute of Technology, SE-100 44 Stockholm, Sweden. Email: tony@kth.se

Abstract—**This supplement contains theoretical material for the paper Lindeberg (2021) "Normative theory of visual receptive fields" published in Heliyon.**

Appendix A gives an explicit proof of the property that the affine Gaussian kernel satisfies the affine diffusion equation.

Appendix B derives the generic form of spatio-temporal smoothing kernel from theoretical necessity results with an explicit parameterization of Galilean transformations.

Appendices C–H give explicit transformation properties of the purely spatial receptive field model and the genuinely spatio-temporal receptive field model under spatial scaling transformations, spatial affine transformations, Galilean transformations and temporal scaling transformations.

Finally, Appendix I describes how true invariance properties can be obtained from the provably covariant receptive field models derived in the main paper, regarding spatial scale invariance, temporal scale invariance, spatial affine invariance and Galilean invariance.

Appendix

A. *Proof that the image representation obtained by convolution with the affine Gaussian kernel satisfies the affine diffusion equation*

In this appendix, we give an elementary direct proof that the solution of the affine diffusion equation (Equation (18) in the main paper)

$$\partial_s L = \frac{1}{2} \nabla^T (\Sigma \nabla L) - \delta^T \nabla L \tag{46}$$

with initial condition $L(x_1, x_2;\ 0) = f(x_1, x_2)$ is given by convolution with affine Gaussian kernels

$$L(x;\ \Sigma_s, \delta_s) = \int_{\xi \in \mathbb{R}^2} g(\xi;\ \Sigma_s, \delta_s)\, f(x - \xi)\, d\xi \tag{47}$$

with

$$g(x;\ \Sigma_s, \delta_s) = \frac{1}{2\pi \sqrt{\det \Sigma_s}}\, e^{-(x-\delta_s)^T \Sigma_s^{-1} (x-\delta_s)/2}, \tag{48}$$

where $\Sigma_s = s\, \Sigma$ and $\delta_s = s\, \delta$.

We start by showing this result in the special case when $\Sigma = I$ (the identity matrix) and $\delta = 0$. Then, the affine diffusion equation (46) reduces to the isotropic diffusion equation

$$\partial_s L = \frac{1}{2} \nabla^T \nabla L = \frac{1}{2} (L_{x_1 x_1} + L_{x_2 x_2}) \tag{49}$$

and the affine Gaussian kernel reduces to the isotropic Gaussian kernel

$$g(x;\ s) = \frac{1}{2\pi s}\, e^{-x^T x/2s} = \frac{1}{2\pi s}\, e^{-(x_1^2 + x_2^2)/2s}. \tag{50}$$

A first observation that we can make is that since the differentiation operators with respect to space $\nabla^T = (\partial_{x_1}, \partial_{x_2})^T$ and with respect to scale ∂_s commute with the convolution operation, it is sufficient to show that the isotropic Gaussian kernel $g(x;\ s)$ (50) satisfies the isotropic diffusion equation (49).

By differentiating the Gaussian kernel with respect to the spatial coordinates, we obtain

$$\partial_{x_1} g(x;\ s) = -\frac{x_1}{s}\, g(x;\ s) \tag{51}$$

$$\partial_{x_2} g(x;\ s) = -\frac{x_2}{s}\, g(x;\ s) \tag{52}$$

and

$$\partial_{x_1 x_1} g(x;\ s) = \frac{(x_1^2 - s)}{s^2}\, g(x;\ s) \tag{53}$$

$$\partial_{x_2 x_2} g(x;\ s) = \frac{(x_2^2 - s)}{s^2}\, g(x;\ s), \tag{54}$$

which gives

$$\begin{aligned} \nabla^T \nabla g(x;\ s) &= g_{x_1 x_1}(x;\ s) + g_{x_2 x_2}(x;\ s) \\ &= \frac{(x_1^2 + x_2^2 - 2s)}{s^2}\, g(x;\ s). \end{aligned} \tag{55}$$

By differentiating the Gaussian kernel with respect to the scale parameter s, we in turn get

$$\begin{aligned} g_s(x;\ s) &= \partial_s \left(\frac{1}{2\pi s}\, e^{-(x_1^2 + x_2^2)/2s} \right) \\ &= \frac{1}{2\pi s}\, e^{-(x_1^2 + x_2^2)/2s} \left(-\frac{x_1^2 + x_2^2}{2} \right) \left(-\frac{1}{s^2} \right) \\ &\quad + \left(-\frac{1}{2\pi s^2} \right) e^{-(x_1^2 + x_2^2)/2s} \\ &= \frac{1}{2\pi s} \left(\frac{x_1^2 + x_2^2}{2 s^2} - \frac{1}{s} \right) e^{-(x_1^2 + x_2^2)/2s} \\ &= \frac{1}{2} \frac{(x_1^2 + x_2^2 - 2 s^2)}{s^2}\, g(x;\ s) \end{aligned} \tag{56}$$

By comparing Equations (55) and (56), we see that that the isotropic Gaussian kernel $g(x;\ s)$ satisfies the isotropic diffusion equation

$$\partial_s g = \frac{1}{2} \nabla^T \nabla g = \frac{1}{2} (g_{x_1 x_1} + g_{x_2 x_2}), \tag{57}$$

which in turn means that image data $f(x_1, x_2)$ that are convolved with the isotropic Gaussian kernel satisfy the isotropic diffusion equation (50).

In a second stage, we consider the transformation property of the diffusion equation and the Gaussian kernel under a two-dimensional affine transformation of the spatial image domain

$$y = Ax. \tag{58}$$

with $A = \begin{pmatrix} a_{11} & a_{12} \\ a_{21} & a_{22} \end{pmatrix}$.

From properties of a two-dimensional change of variables, if follows that the gradient operators in the two domains are then related according to (where we now subscript the gradient operator ∇ with respect to the domain x or y it is defined over)

$$\nabla_X = \begin{pmatrix} \partial_{x_1} \\ \partial_{x_2} \end{pmatrix} = A^T \begin{pmatrix} \partial_{y_1} \\ \partial_{y_2} \end{pmatrix} = A^T \nabla_Y. \quad (59)$$

Thus, the diffusion term in the isotropic diffusion equation (49) transforms according to

$$\begin{aligned} \nabla_X^T \nabla_X L &= (A^T \nabla_Y)^T (A^T \nabla_Y) L \\ &= \nabla_Y^T (A A^T) \nabla_Y L = \nabla_Y^T \Sigma \nabla_Y L \end{aligned} \quad (60)$$

for $\Sigma = A A^T$.

if we assume that two image patterns $f_X(x)$ and $f_Y(y)$ are related according to the affine transformation (58) such that $f_X(x) = f_Y(y)$ for $y = Ax$, then the convolution operation with respect to an isotropic Gaussian kernel

$$L_X(x;\; s) = \int_{\xi \in \mathbb{R}^2} g(\xi;\; s)\, f_X(x - \xi)\, d\xi \quad (61)$$

by the change of variables $\eta = A\xi$ with $d\eta = |\det A|\, d\xi$ and the corresponding transformation of the Gaussian kernel

$$\begin{aligned} g(\xi;\; s) &= \frac{1}{2\pi s}\, e^{-\xi^T \xi / 2s} = \frac{1}{2\pi s}\, e^{-(A^{-1}\eta)^T (A^{-1}\eta)/2s} \\ &= \frac{1}{2\pi s}\, e^{-\eta^T A^{-T} A^{-1} \eta / 2s} = \frac{1}{2\pi s}\, e^{-\eta^T \Sigma^{-1} \eta / 2s} \end{aligned} \quad (62)$$

for $\Sigma = A A^T$ with $\Sigma^{-1} = A^{-T} A^{-1}$ and $|\det A| = \sqrt{|\det \Sigma|} = \sqrt{\det \Sigma}$ transforms according to

$$\begin{aligned} L_X(x;\; s) &= \int_{\eta \in \mathbb{R}^2} \frac{1}{2\pi s \sqrt{\det \Sigma}}\, e^{-\eta^T \Sigma^{-1} \eta / 2s} f_Y(y - \eta)\, d\eta \\ &= \int_{\eta \in \mathbb{R}^2} g(\eta;\; s\,\Sigma)\, f_Y(y - \eta)\, d\eta = L_Y(y;\; s\Sigma), \end{aligned} \quad (63)$$

where the subscripts X or Y to the smoothed intensity values L_X denote which domains the functions are defined over.

This result, in combination with the transformation property of the Laplacian operator (60), implies that convolution with of the transformed image pattern $f_Y(y)$ with the affine Gaussian kernel

$$g_Y(y;\; s\,\Sigma) = \frac{1}{2\pi \sqrt{\det s\Sigma}}\, e^{-y^T (s\Sigma^{-1}) y/2} \quad (64)$$

without a spatial drift term δ satisfies the affine diffusion equation without a drift term

$$\partial_s L = \frac{1}{2} \nabla_Y^T \left(\Sigma \nabla_Y L \right). \quad (65)$$

Let us finally introduce a drift term by performing the following change of variables

$$z = y + \delta\, s \quad (66)$$

$$s' = s \quad (67)$$

in this equation. By properties of a change of variables, it follows that the derivative operators transform according to

$$\nabla_Y = \begin{pmatrix} \partial_{y_1} \\ \partial_{y_2} \end{pmatrix} = \begin{pmatrix} \partial_{z_1} \\ \partial_{z_2} \end{pmatrix} = \nabla_Z, \quad (68)$$

$$\partial_s = \delta^T \nabla_Y L + \partial_{s'}. \quad (69)$$

By replacing ∂_s and ∇_Y in (65) with these expressions, it follows that (65) transforms into

$$\partial_{s'} L = \frac{1}{2} \nabla_Z^T \left(\Sigma \nabla_Z L \right) - \delta^T \nabla_Z L. \quad (70)$$

By correspondingly replacing replacing $y = z - \delta\, s$ in (63) and (71), it follows that convolution with affine Gaussian kernels with a drift term

$$g_Z(y;\; s\,\Sigma, s\,\delta) = \frac{1}{2\pi \sqrt{\det s\Sigma}}\, e^{-(z-\delta s)^T (s\Sigma)^{-1} (z - \delta s)/2} \quad (71)$$

satisfies the affine diffusion equation (70) with a drift term, which proves the result.

Alternatively, the fact that convolution (47) with affine Gaussian kernels of the form (48) satisfies the diffusion equation (46), can also be shown by explicitly differentiating the affine Gaussian kernel with respect to the spatial variables $x = (x_1, x_2)$ and scale s. Because of complexity of the calculations, these calculations are then easiest done in a package for symbolic manipulation, such as Mathematica or Maple.

B. Derivation of the generic form of spatio-temporal smoothing kernels (in Equation (24) in the main paper)

In this appendix, we will describe the logical steps in the derivation that leads to the form of spatio-temporal smoothing kernels in Equation (24) in the main paper):

$$T(x_1, x_2, t;\; s, \tau;\; v, \Sigma) = g(x_1 - v_1 t, x_2 - v_2 t;\; s\,\Sigma)\, h(t;\; \tau). \quad (72)$$

1) The case of a non-causal temporal domain: Let us consider the joint spatio-temporal domain, and denote coordinates in this domain by $p = (x_1, x_2, t)^T$. Let us assume the conditions of linearity and shift invariance in combination with a semi-group structure over a continuous scale parameter s that obeys non-enhancement of local extrema. Then, from similar logical steps[9] in the derivation of the diffusion equation over a two-dimensional purely spatial domain (46), it follows that the internal spatio-temporal scale-space representation L must satisfy a diffusion equation over the joint spatio-temporal domain

$$\partial_s L = \frac{1}{2}\, \nabla_P^T \left(\Sigma \nabla_P L \right) - \delta^T \nabla_P L \quad (73)$$

with the differentiation operator over joint space-time of the form $\nabla_P^T = (\partial_{x_1}, \partial_{x_2},\; \partial_t)^T$. In terms of convolution kernels, the solution of (73) is given by convolution over joint space-time

$$L(p;\; \Sigma_s, \delta_s) = \int_{\xi \in \mathbb{R}^3} g_{3D}(\xi;\; \Sigma_s, \delta_s)\, f(p - \xi)\, d\xi \quad (74)$$

[9]The combination of Lemma 2 and Theorem 5 in [1] over a three-dimensional spatio-temporal domain.

with three-dimensional spatio-temporal Gaussian kernels of the form

$$g_{3D}(p;\ \Sigma_s, \delta_s) = \frac{1}{2\pi\sqrt{\det \Sigma_s}}\, e^{-(p-\delta_s)^T \Sigma_s^{-1} (p-\delta_s)/2}, \tag{75}$$

where $\Sigma_s = s\,\Sigma$ and $\delta_s = s\,\delta$. For simplicity, we henceforth assume $\delta = 0$ implying that also $\delta_s = 0$, which is a relevant assumption in a monocular setting. In a binocular setting, we could use the translational offset δ for modelling the disparity between the two eyes.

Consider next a Galilean transformation $x' = x + ut$ of joint space-time, where $u = (u_1, u_2)^T$ is the image velocity,

$$x_1' = x_1 + u_1 t, \tag{76}$$
$$x_2' = x_2 + u_2 t, \tag{77}$$
$$t_1' = t, \tag{78}$$

on matrix form written as $p' = Gp$, with the Galilean transformation matrix G of the form

$$G = \left(\begin{array}{ccc} 1 & 0 & u_1 \\ 0 & 1 & u_2 \\ 0 & 0 & 1 \end{array} \right). \tag{79}$$

Under a general affine transformation of joint space-time, $p' = Ap$, we have that the spatio-temporal covariance matrix Σ in the convolution integral (75) and the diffusion equation (73) transforms according to

$$\Sigma' = A\,\Sigma\,A^T. \tag{80}$$

This transformation property is similar to the transformation property in the two-dimensional case as described in Appendix D. It holds in arbitrary dimensions and can be proven by a change of variables in the convolution integral, as done in [2, Section 15.3.1] or [3, Section 4.1]

If we assume that the spatio-temporal covariance matrix Σ over the joint spatio-temporal domain is of the form

$$\Sigma = \left(\begin{array}{ccc} \Sigma_{11} & \Sigma_{12} & 0 \\ \Sigma_{12} & \Sigma_{22} & 0 \\ 0 & 0 & \tau \end{array} \right), \tag{81}$$

where the submatrix $\Sigma_{space} = \left(\begin{array}{cc} \Sigma_{11} & \Sigma_{12} \\ \Sigma_{12} & \Sigma_{22} \end{array} \right)$ represents a purely spatial covariance matrix describing the amount of spatial smoothing and τ the amount of temporal smoothing, then it follows that the spatio-temporal covariance matrix over the Galilean transformed frame parameterized by $p' = Gp$ is given by

$$\Sigma' = G\,\Sigma\,G^T. \tag{82}$$

By inserting G from (79) and Σ from (81) in (82) and performing the matrix multiplications explicitly, we obtain

$$\Sigma' = \left(\begin{array}{ccc} \Sigma_{11} + u_1^2\tau & \Sigma_{12} + u_1 u_2 \tau & u_1\tau \\ \Sigma_{12} + u_1 u_2 \tau & \Sigma_{22} + u_2^2\tau & u_2\tau \\ u_1\tau & u_2\tau & \tau \end{array} \right). \tag{83}$$

This relationship means that a three-dimensional spatio-temporal covariance matrix Σ' can be parameterized[10] as the combination of a two-dimensional purely spatial covariance matrix Σ_{space} in combination with a Galilean transformation represented by a Galilean transformation matrix G and a purely temporal amount of smoothing τ.

In terms of convolution kernels, this relationship means that the three-dimensional Gaussian kernel g_{3D} in (75) is decomposed into the combination of a two-dimensional Gaussian kernel over image space and a one-dimensional Gaussian kernel over time

$$g_{3D}(p;\ \Sigma_{space-time}) = g_{2D}(x - ut;\ \Sigma_{space})\, g_{1D}(t;\ \tau). \tag{84}$$

This property follows since with a space-time separable spatio-temporal covariance matrix of the form (81), we have that the argument of the exponential function in the three-dimensional Gaussian kernel over joint space-time (75) is given by

$$p'^T \Sigma_{space-time}^{-1} p/2 = (x'^T \Sigma_{space}^{-1} x + t^2/\tau)/2, \tag{85}$$

implying a decomposition of the three-dimensional space-time separable spatio-temporal Gaussian kernel of the form

$$g_{3D}(p;\ \Sigma_{space-time}) = g_{2D}(x;\ \Sigma_{space})\, g_{1D}(t;\ \tau), \tag{86}$$

which is then combined with a Galilean transformation (79) to give the form (84).

Thus, in the case of non-causal time, the spatio-temporal smoothing kernels should be of the form

$$T(x_1, x_2, t;\ s, \tau;\ v, \Sigma) = g(x_1 - v_1 t, x_2 - v_2 t;\ s\,\Sigma)\, h(t;\ \tau) \tag{87}$$

with the spatial smoothing kernel g being a two-dimensional Gaussian kernel and the temporal kernel h being a one-dimensional Gaussian kernel.

2) The case of a time-causal temporal domain: For the case of a time-causal temporal domain, an axiomatic derivation based on a somewhat different assumptions over an $N+1$-dimensional spatio-temporal domain leads to temporal smoothing kernels of the form [1, Equation (91)]

$$T(x, t;\ \Sigma, v, \tau) = g(x - vt;\ \Sigma)\, \phi(t;\ \tau) \tag{88}$$

where g is a spatial Gaussian smoothing kernel with its spatial shape determined by the spatial covariance matrix Σ and ϕ is a temporal smoothing kernel with its temporal extent determined by the parameter τ.

In the case of a two-dimensional domain, this expression can specifically be written

$$T(x_1, x_2, t;\ \Sigma, v, \tau) = g(x_1 - v_1 t, x_2 - v_2 t;\ \Sigma)\, \phi(t;\ \tau), \tag{89}$$

[10]Given a spatio-temporal covariance matrix Σ' with elements Σ'_{ij} for $i, j \in \{1, 2, 3\}$, we can from (83) determine the parameters τ, u and Σ_{space} in the Galilean parameterization according to $\tau = \Sigma'_{33}$, $u_1 = \Sigma'_{13}/\tau = \Sigma'_{13}/\Sigma'_{33}$, $u_2 = \Sigma'_{23}/\tau = \Sigma'_{23}/\Sigma'_{33}$, $\Sigma_{11} = \Sigma'_{11} - u_1^2\tau = \Sigma'_{11} - {\Sigma'_{13}}^2/\Sigma'_{33}$, $\Sigma_{12} = \Sigma'_{12} - u_1 u_2 \tau = \Sigma'_{12} - \Sigma'_{13}\Sigma'_{23}/\Sigma'_{33}$ and $\Sigma_{22} = \Sigma'_{22} - u_2^2\tau = \Sigma'_{22} - {\Sigma'_{23}}^2/\Sigma'_{33}$.

which is notably also of a similar form as (87), while for a different temporal smoothing kernel.

Motivated by the fact that two independent axiomatic derivations lead to a formally similar form of spatio-temporal smoothing kernels, while using different temporal smoothing functions, we will in this section complement those treatments by replacing the temporal smoothing kernels in (87) and (89) by another form of temporal smoothing kernel that is determined from other assumptions and has better temporal dynamics properties in the time-causal case than the temporal kernel ϕ in (89).

The assumption that we will start from is to require that when a purely temporal signal is convolved with a temporal kernel h, then this operation must not increase the number of local extrema, or equivalently the number of zero-crossings, in the signal. Interestingly, such kernels can be completely classified.

A theoretical result by Schoenberg [4] (see [5, Section 3.2] for an overview) implies that for one-dimensional continuous signals there are four primitive types of linear and shift-invariant smoothing transformations that are variation-diminishing in this sense: convolution with the *Gaussian kernel,*

$$h(\xi) = e^{-\gamma \xi^2}, \tag{90}$$

convolution with *truncated exponential functions,*

$$h(\xi) = \begin{cases} e^{-|\lambda|\xi} & \xi \geq 0, \\ 0 & \xi < 0, \end{cases} \quad h(\xi) = \begin{cases} e^{|\lambda|\xi} & \xi \leq 0, \\ 0 & \xi > 0, \end{cases} \tag{91}$$

as well as trivial *translations* and *rescalings*. Moreover, it means that a shift-invariant linear transformation is variation diminishing in this sense if and only if it can be decomposed into these primitive operations.

Thus, kernels that can be decomposed into a cascade of truncated exponential kernels are the only non-trivial time-causal temporal smoothing kernels that guarantee that the number of local extrema, or equivalently the number of zero-crossings, in the signal must not increase under a temporal convolution operation.

In this way, we are lead to consider spatio-temporal smoothing kernels of the form

$$T(x_1, x_2, t;\ s, \tau;\ v, \Sigma) = g(x_1 - v_1 t, x_2 - v_2 t;\ s\,\Sigma)\, h(t;\ \tau), \tag{92}$$

with the temporal smoothing kernel h being a composition of a set of truncated exponential kernels coupled in cascade, possibly having different time constants $\mu = 1/|\lambda|$.

In terms of physical models, repeated convolution with truncated exponential kernels over time corresponds to coupling a series of *first-order integrators* with time constants μ_k in cascade

$$\partial_t L(t;\ \tau_k) = \frac{1}{\mu_k} \left(L(t;\ \tau_{k-1}) - L(t;\ \tau_k) \right) \tag{93}$$

with $L(t;\ 0) = f(t)$. Computationally, such operations are highly efficient and admit for direct implementation in wetware that emulates first-order integration over time, and where the temporal scale levels together also serve as a sufficient time-recursive memory of the past. Thus, these computational operations are also *time-recursive*.

C. Transformation property of spatial image representations under spatial scaling transformations

Consider two images $f(x)$ and $f'(x')$, with $x = (x_1, x_2)^T$ and $x' = (x_1', x_2')^T$, that are related by a uniform scaling transformation, such that

$$f'(x') = f(x) \quad \text{for} \quad x' = Sx, \tag{94}$$

where $S > 0$ is a spatial scaling factor.

Consider the spatial scale-space representations L and L that are obtained by convolving the images $f(x)$ and $f'(x')$, respectively, by kernels T and T' of the form in Equation (22) in the main paper

$$T(x;\ s, \Sigma) = g(x;\ s\,\Sigma) = \frac{1}{2\pi s \sqrt{\det \Sigma}}\, e^{-x^T \Sigma^{-1} x/2s}, \tag{95}$$

with $T(x;\ s, \Sigma) = g(x;\ s\,\Sigma)$ and $T(x';\ s', \Sigma) = g(x';\ s'\,\Sigma)$, respectively, Then, the resulting spatial scale-space representations over the two mutually rescaled domains are related according to

$$L'(x';\ s') = L(x;\ s) \quad \text{for} \quad s' = S^2 s. \tag{96}$$

In this sense, the solutions of (46) obey *spatial scale covariance*, as illustrated in the commutative diagram in Figure 21, which makes it possible for the family of receptive field responses to consistently handle variations in image structures caused by objects of different size in the world and objects at different distances to the observer.

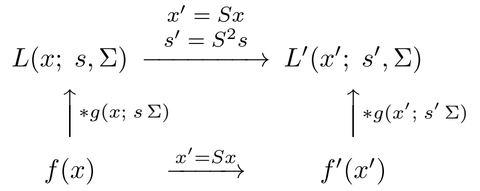

Fig. 21. Commutative diagram for receptive field responses under *uniform scaling transformations* of the spatial domain. Such a spatial rescaling transformation may, for example, represent images of similar objects of different size or objects that are observed with different distances between the camera and the object. (The commutative diagram should be read from the lower left corner to the upper right corner, and means that irrespective of whether the image is first convolved with a Gaussian kernel and then scaled, or whether the image is first scaled and then convolved with another Gaussian kernel, we get the same result provided that the values of the scale parameters s and s' are properly matched to the magnitude S of the scaling transformation.)

D. Transformation property of spatial image representations under spatial affine transformations

Consider two images $f_L(x_L)$ and $f_R(x_R)$, with $x_L = (x_{L1}, x_{L2})^T$ and $x_R = (x_{R1}, x_{R2})^T$, that are related by an affine transformation, such that

$$f_L(x_L) = f_R(x_R) \quad \text{for} \quad x_R = A x_L, \tag{97}$$

where A is a non-singular affine transformation matrix $A = \begin{pmatrix} a_{11} & a_{12} \\ a_{21} & a_{22} \end{pmatrix}$.

Consider the spatial scale-space representations L_L and L_R that are obtained by convolving the images f_L and f_R, respectively, by kernels T_L and T_R of the form (95), with

$T_L(x_L;\ s, \Sigma_L) = g(x_L;\ s\,\Sigma_L)$ and $T_R(x_R;\ s, \Sigma_R) = g(x_R;\ s\,\Sigma_R)$.

Then, the resulting spatial scale-space representations the two mutually affine related domains are related according to

$$L_L(x_L;\ s, \Sigma_L) = L_R(x_R;\ s, \Sigma_R) \quad \text{for} \quad \Sigma_R = A \Sigma_L A^T, \tag{98}$$

see [2, Section 15.3.1] or [3, Section 4.1].

In this sense, the solutions of (46) obey *spatial affine covariance*, as illustrated in the commutative diagram in Figure 22, which makes it possible for the family of receptive field responses to consistently handle variations in the projected shape of surface structures caused by variations in the slant and the tilt angles of a local surface patch in relation to the visual observer.

$$\begin{array}{ccc} L_L(x_L;\ s, \Sigma_L) & \xrightarrow[]{\substack{x_R = A x_L \\ \Sigma_R = A \Sigma_L A^T}} & L_R(x_R;\ s, \Sigma_R) \\ \Big\uparrow {\scriptstyle *g(x_L;\ s\,\Sigma_L)} & & \Big\uparrow {\scriptstyle *g(x_R;\ s\,\Sigma_R)} \\ f_L(x_L) & \xrightarrow{x_R = A x_L} & f_R(x_R) \end{array}$$

Fig. 22. Commutative diagram for receptive field responses under *affine transformations* of the spatial domain. Such an affine scaling transformation may, for example, represent a local linearization of the perspective mapping of a local surface patch, leading to a foreshortening transformation in combination with local rotations and a scaling transformation. (The commutative diagram should be read from the lower left corner to the upper right corner, and means that irrespective of whether the image is first convolved with an affine Gaussian kernel and then subject to an affine transformation, or whether the image is first affine transformed and then convolved with another affine Gaussian kernel, we get the same result provided that the covariance matrices Σ_L and Σ_R are properly matched to the affine transformation A.)

E. Transformation property of spatio-temporal image representations under spatial scaling transformations

Consider two spatio-temporal image patterns $f(x_1, x_2, t)$ and $f'(x_1', x_2', t)$ that are related by a spatial scaling transformation, such that

$$f'(x_1', x_2', t) = f(x_1, x_2, t) \tag{99}$$

for

$$x_1' = S x_1 \quad \text{and} \quad x_2' = S x_2, \tag{100}$$

where $S > 0$ is a spatial scaling factor.

Let $L(x_1, x_2, t;\ s, \tau;\ v, \Sigma)$ and $L'(x_1', x_2', t;\ s', \tau;\ v', \Sigma)$ be the smoothed spatio-temporal image representations obtained by convolving the patterns $f(x_1, x_2, t)$ and $f'(x_1', x_2', t)$, respectively, by kernels of the form (72) with $T(x_1, x_2, t;\ s, \tau;\ v, \Sigma) = g(x_1 - v_1 t, x_2 - v_2 t;\ s\,\Sigma)\, h(t;\ \tau)$ and $T'(x_1', x_2', t;\ s', \tau;\ v', \Sigma) = g(x_1' - v_1' t, x_2' - v_2' t;\ s'\,\Sigma)\, h(t;\ \tau)$, respectively.

Then, the spatio-temporal scale-space representations are related according to

$$L(x_1, x_2, t;\ s, \tau;\ v, \Sigma) = L'(x_1', x_2', t;\ s', \tau;\ v', \Sigma) \tag{101}$$

for

$$s' = S^2 s \quad \text{and} \quad v' = S v. \tag{102}$$

In this sense, the resulting spatio-temporal image representations obey *spatial scale covariance*, as illustrated in the commutative diagram in Figure 23, which makes it possible for the family of receptive field responses to consistently handle variations in image structures caused by objects of different size in the world and objects at different distances to the observer.

$$\begin{array}{ccc} L(x, t;\ s, \tau;\ v, \Sigma) & \xrightarrow[]{\substack{x' = Sx \\ s' = S^2 s \\ v' = Sv}} & L'(x', t;\ s', \tau;\ v', \Sigma) \\ \Big\uparrow {\scriptstyle *T(x, t;\ s, \tau;\ v, \Sigma)} & & \Big\uparrow {\scriptstyle *T(x', t;\ s', \tau;\ v', \Sigma)} \\ f(x, t) & \xrightarrow{x' = Sx} & f'(x', t) \end{array}$$

Fig. 23. Commutative diagram for receptive field responses under *spatial scaling transformations* of the spatio-temporal image domain. Such a spatial rescaling transformation may, for example, represent images of similar objects of different size or objects that are observed with different distances between the camera and the object. (The commutative diagram should be read from the lower left corner to the upper right corner, and means that irrespective of whether the image is first convolved with a Gaussian kernel and then scaled, or whether the image is first scaled and then convolved with another Gaussian kernel, we get the same result provided that the values of the spatial scale parameters s and s' and the velocity values v and v' are properly matched to the magnitude S of the scaling transformation.)

F. Transformation property of spatio-temporal image representations under spatial affine transformations

Consider two spatio-temporal image patterns $f_L(x_L, t)$ and $f_R(x_R, t)$, with $x_L = (x_{L1}, x_{L2})^T$ and $x_R = (x_{R1}, x_{R2})^T$, that are related by an affine transformation, such that

$$f_L(x_L, t) = f_R(x_R, t) \quad \text{for} \quad x_R = A x_L, \tag{103}$$

where A is a non-singular affine transformation matrix $A = \begin{pmatrix} a_{11} & a_{12} \\ a_{21} & a_{22} \end{pmatrix}$.

Let $L_L(x_L, t;\ s, \tau;\ v_L, \Sigma_L)$ and $L_R(x_R, t;\ s, \tau;\ v_R, \Sigma_R)$ be the smoothed spatio-temporal image representations obtained by convolving the patterns $f(x_L, t)$ and $f'(x_R, t)$, respectively, by kernels of the form (72) with $T_L(x_L, t;\ s, \tau;\ v_L, \Sigma_L) = g(x_L - v_L t;\ s\,\Sigma_L)\, h(t;\ \tau)$ and $T_R(x_R, t;\ s, \tau;\ v_R, \Sigma_R) = g(x_R - v_R t;\ s\,\Sigma_R)\, h(t;\ \tau)$, respectively.

Then, the spatio-temporal scale-space representations are related according to

$$L_L(x_L, t;\ s, \tau;\ v_L, \Sigma_L) = L_R(x_R, t;\ s, \tau;\ v_R, \Sigma_R) \tag{104}$$

for

$$\Sigma_R = A \Sigma_L A^T \quad \text{and} \quad v_R = A v_L. \tag{105}$$

In this sense, the spatio-temporal image representations obey *spatial affine covariance*, as illustrated in the commutative diagram in Figure 24, which makes it possible for the family of receptive field responses to consistently handle variations in the projected shape of surface structures caused by variations in the slant and the tilt angles of a local surface patch in relation to the visual observer.

$$
\begin{array}{ccc}
L_L(x_L, t;\ s, \tau;\ v_L, \Sigma_L) & \xrightarrow[]{\substack{x_R = A x_L \\ \Sigma_R = A \Sigma_L A^T \\ v_R = A v_L}} & L_R(x_R, t;\ s, \tau;\ v_R, \Sigma_R) \\
\Big\uparrow {\scriptstyle *T(x_L, t;\ s, \tau;\ v_L, \Sigma_L)} & & \Big\uparrow {\scriptstyle *T(x_R, t;\ s, \tau;\ v_R, \Sigma_R)} \\
f_L(x_L, t) & \xrightarrow{x_R = A x_L} & f_R(x_R, t)
\end{array}
$$

Fig. 24. Commutative diagram for receptive field responses under *spatial affine transformations* of the spatio-temporal image domain. Such an affine scaling transformation may, for example, represent a local linearization of the perspective mapping of a local surface patch, leading to a foreshortening transformation in combination with local rotations and a scaling transformation. (The commutative diagram should be read from the lower left corner to the upper right corner, and means that irrespective of whether the image is first convolved with a Gaussian kernel and then scaled, or whether the image is first scaled and then convolved with another Gaussian kernel, we get the same result provided that the covariance matrices Σ_L and Σ_R and the velocity values v and v' are properly matched to the affine transformation A.)

G. Transformation property of spatio-temporal image representations under Galilean transformations

Consider two spatio-temporal image patterns $f(x_1, x_2, t)$ and $f'(x_1', x_2', t)$ that are related by a Galilean transformation, such that

$$f'(x_1', x_2', t) = f(x_1, x_2, t) \tag{106}$$

for

$$x_1' = x_1 + u_1 t \quad \text{and} \quad x_2' = x_2 + u_2 t, \tag{107}$$

and corresponding to motion with contant relative image velocity $u = (u_1, u_2)^T$ between the two reference frames.

Let $L(x_1, x_2, t;\ s, \tau;\ v, \Sigma)$ and $L'(x_1', x_2', t;\ s, \tau;\ v', \Sigma)$ be the smoothed spatio-temporal scale-space representations obtained by convolving the patterns $f(x_1, x_2, t)$ and $f'(x_1', x_2', t)$, respectively, by kernels of the form (72) with $T(x_1, x_2, t;\ s, \tau;\ v, \Sigma) = g(x_1 - v_1 t, x_2 - v_2 t;\ s\,\Sigma)\, h(t;\ \tau)$ and $T'(x_1', x_2', t;\ s, \tau;\ v', \Sigma) = g(x_1' - v_1' t, x_2' - v_2' t;\ s\,\Sigma)\, h(t;\ \tau)$, respectively.

Then, the spatio-temporal scale-space representations are related according to

$$L(x_1, x_2, t;\ s, \tau;\ v, \Sigma) = \\ = L'(x_1', x_2', t;\ s, \tau;\ v', \Sigma) \tag{108}$$

for

$$v' = u + v. \tag{109}$$

In this sense, the spatio-temporal image representations obtained by convolution with kernels of the form (72) obey *Galilean covariance*, as illustrated in the commutative diagram in Figure 25, which makes it possible for the family of receptive field responses to consistently handle objects and spatio-temporal events in the world with different relative motion in relation to the viewing direction of the observer.

H. Transformation property of spatio-temporal image representations under temporal scaling transformations

Consider two spatio-temporal image patterns $f(x_1, x_2, t)$ and $f'(x_1, x_2, t')$ that are related by a temporal scaling transformation, such that

$$f'(x_1, x_2, t') = f(x_1, x_2, t) \quad \text{for} \quad t' = St \tag{110}$$

for some $S > 0$.

$$
\begin{array}{ccc}
L(x, t;\ s, \tau;\ v, \Sigma) & \xrightarrow[]{\substack{x' = x + ut \\ v' = v + u}} & L'(x', t;\ s, \tau;\ v', \Sigma) \\
\Big\uparrow {\scriptstyle *T(x, t;\ s, \tau;\ v, \Sigma)} & & \Big\uparrow {\scriptstyle *T(x', t;\ s, \tau;\ v', \Sigma)} \\
f(x, t) & \xrightarrow{x' = x + ut} & f'(x', t)
\end{array}
$$

Fig. 25. Commutative diagram for receptive field responses under *Galilean transformations* of the spatio-temporal image domain. Such Galilean transformations describe the effect of observing a visual scene with different relative motion between the viewing direction and the objects or events that are observed. (The commutative diagram should be read from the lower left corner to the upper right corner, and means that irrespective of whether the image is first convolved with a velocity-adapted spatio-temporal smoothing kernel and then subject to a Galilean transformation, or whether the image data arising from a scene is first subject to a Galilean transformation and then convolved with a velocity-adapted spatio-temporal kernel, we get the same result provided that the velocity parameters v and v' are properly matched to the relative motion u between the two spatio-temporal image patterns.)

Assume that the temporal smoothing kernel $h(t;\ \tau)$ in (72) is either a one-dimensional Gaussian kernel in the case of a non-causal temporal domain or the time-causal limit kernel defined from Equation (30) in the main paper

$$\hat{\Psi}(\omega;\ \tau, c) = \prod_{k=1}^{\infty} \frac{1}{1 + i\, c^{-k} \sqrt{c^2 - 1} \sqrt{\tau}\, \omega}. \tag{111}$$

in the case of a time-causal temporal domain.

Let $L(x_1, x_2, t;\ s, \tau;\ v, \Sigma)$ and $L'(x_1, x_2, t';\ s, \tau';\ v', \Sigma)$ be the smoothed spatio-temporal scale-space representations obtained by convolving the patterns $f(x_1, x_2, t)$ and $f'(x_1, x_2, t')$, respectively, by kernels of the form (72) with $T(x_1, x_2, t;\ s, \tau;\ v, \Sigma) = g(x_1 - v_1 t, x_2 - v_2 t;\ s\,\Sigma)\, h(t;\ \tau)$ and $T'(x_1, x_2, t';\ s, \tau';\ v', \Sigma) = g(x_1 - v_1' t', x_2 - v_2' t';\ s\,\Sigma)\, h(t';\ \tau')$, respectively.

Then, the spatio-temporal scale-space representations are related according to

$$L(x_1, x_2, t;\ s, \tau;\ v, \Sigma) = \\ = L'(x_1, x_2, t';\ s, \tau';\ v', \Sigma) \tag{112}$$

for

$$\tau' = S^2 \tau \quad \text{and} \quad v' = v/S. \tag{113}$$

In the case of a non-causal temporal domain, with the temporal smoothing performed by a one-dimensional Gaussian kernel, this relationship holds for any S.

In the case of a time-causal temporal domain, with the temporal smoothing performed by the time-causal limit kernel, this relationship holds only for temporal scaling factors S that are integer powers of the distribution parameter c in the time-causal limit kernel, *i.e.,* $S = c^j$ for integer values of j, see [5, Equation (49)].

The reason why the temporal scaling relation holds only for a discrete set of temporal scaling factors is that the temporal scale levels τ_k defined from a set of truncated exponential kernels coupled in cascade are genuinely discrete. For temporal scaling factors in between these discrete values, the temporal scaling transformation will be an approximation, with the accuracy depending on how densely the temporal scale values are sampled, as determined by the distribution parameter c.

In this sense, the spatio-temporal scale-space representations obtained by convolution with kernels of the form (72) will either fully obey or numerically approximate *temporal scale covariance*, as illustrated in the commutative diagram in Figure 26. This makes it possible for the family of receptive field responses to consistently handle spatio-temporal events that occur with different speed—faster or slower.

$$\begin{array}{ccc}
L(x,t;\; s,\tau;\; v,\Sigma) & \xrightarrow[v' = v/S]{t' = St,\; \tau' = S^2\tau} & L'(x,t';\; s,\tau';\; v',\Sigma) \\
\big\uparrow {\scriptstyle *T(x,t;\; s,\tau;\; v,\Sigma)} & & \big\uparrow {\scriptstyle *T(x,t';\; s,\tau';\; v',\Sigma)} \\
f(x,t) & \xrightarrow{t'=St} & f'(x,t')
\end{array}$$

Fig. 26. Commutative diagram for receptive field responses under *temporal scaling transformations* of the spatio-temporal image domain. Such transformations describe the effect of events occurring slower or faster. (The commutative diagram should be read from the lower left corner to the upper right corner, and means that irrespective of whether the image is first convolved with a velocity-adapted spatio-temporal smoothing kernel and then subject to temporal scaling transformation, or whether the image data arising from a scene is first subject to a temporal scaling transformation and then convolved with a velocity-adapted spatio-temporal kernel, we get the same result provided that the velocity parameters temporal scale parameters τ and τ' and the velocity parameters v and v' are properly matched to the relative temporal scaling factor S between the two spatio-temporal image patterns.)

I. How covariant receptive fields at lower layers in the visual hierarchy enable invariances at higher levels in the visual hierarchy.

Covariance of visual receptive fields means that if we have a family of receptive fields that are covariant with respect to a family of image transformations, then under transformations of the input to the visual receptive fields, it is always possible to find a member in the receptive field family, such that the output from the receptive fields can be matched before and after the image transformation.

In this appendix section, we outline mathematical arguments for how this logical structure of covariant receptive fields enables the computation of visual invariances at higher levels in the visual hierarchy. This material constitutes a theoretically updated description of the principles underlying the framework for achieving invariant visual operations based on receptive field responses outlined in [6].

In brief, spatial scale invariance and temporal scale invariance can be obtained by choosing receptive field responses from the spatial and temporal scale levels that maximize scale-normalized receptive field responses over variations of spatial and temporal scales, respectively. Invariance to spatial affine transformations can be obtained by groups of spatial receptive fields that measure the similarity of the receptive field responses with respect to an internal spatial model, below formalized in terms of an affine invariant fixed-point property that leads to an affine invariant reference frame. Invariance to Galilean transformation can be achieved by groups of receptive fields that measure the similarity of the receptive field responses with respect to an internal spatio-temporal model, below formalized in terms of the notion of Galilean diagonalization that leads to a Galilean invariant reference frame.

In the following, we describe how such operations can be performed in more detail, in ways that have been demonstrated to give highly useful results for computer vision operations that implement visual invariances.

1) Spatial scale invariance: Let us first focus on spatial scaling transformations. In [7], [8], [9] a framework for spatial scale selection is described based on detecting local extrema over scale of scale-normalized spatial derivatives of the form

$$\partial_{\xi_1} = s^{\gamma_s/2}\, \partial_{x_1}, \quad \partial_{\xi_2} = s^{\gamma_s/2}\, \partial_{x_2}, \qquad (114)$$

where $\gamma_s > 0$, and corresponding to scale-normalized visual receptive fields of the form in Equation (31) in the main paper

$$T_{\varphi^{m_1}\perp\varphi^{m_2},norm}(x_1, x_2;\; s, \Sigma) = s_{\varphi}^{m_1\gamma_s/2}\, s_{\perp\varphi}^{m_2\gamma_s/2}\, \partial_{\varphi}^{m_1} \partial_{\perp\varphi}^{m_2} \left(g(x_1, x_2;\; s\Sigma)\right), \quad (115)$$

with the scale parameters being equal in the isotropic (non-affine) case $s_{\varphi} = s_{\perp\varphi} = s$.

Specifically, theoretical results in [7], [8], [9] show that: (i) local extrema over spatial scales of such scale-normalized spatial derivatives computed from the internal spatial scale-space representation L will for suitable combinations of derivative responses (as made precise in the papers) reflect a characteristic length of the image structures that gave result rise to the feature responses and (ii) transform in scale-covariant way such that if a feature detector responds by a maximum over spatial scales at some position x_0 and some scale s_0 for an input image f, then for a rescaled input image $f'(x') = f(x)$ with $x' = Sx$, there will be a maximum over spatial scales at position $x'_0 = Sx_0$ and at scale $s'_0 = S^2 s_0$.

This theoretical result holds for any value of $\gamma_s > 0$. If $\gamma_s = 1$, then the scale-normalized magnitude values at the extrema over spatial scales are additionally equal.

If we in turn normalize the local image patterns f and f' by rescaling transformations around the points x_0 and x'_0, respectively, by their respective scale parameters s_0

and s'_0, then the rescaled frames $f_{ref}(y) = f(x)$ for $y = x/\sqrt{s_0}$ and $f'_{ref}(y') = f'(x')$ for $y' = x'/\sqrt{s'_0}$ will be equal $f'_{ref}(y') = f_{ref}(y)$ for $y' = y$. This means that any image measurement that is expressed in the scale-normalized frame will be invariant under spatial scaling transformations. (In practice, it is not necessary to perform explicit spatial warping between the two frames—it is sufficient to formulate computational operations that can equivalently be related to such scale-invariant reference frames.) Thus, detection of local extrema over scales of scale-normalized derivatives constitutes a general mechanism for scale selection and for obtaining temporal scale invariance.

In the area of computer vision, this principle has found numerous application for making classical computer visions robust under scaling transformations [7], [8], [10], [11], [12], [13], [14], [15], [16], [9]. In a biological architecture, corresponding operations could be implemented in wetware based on multiple receptive fields over different sizes in the visual domain and an operation that selects the maximum value of the responses in combination with a routing mechanism.

2) Temporal scale invariance: Corresponding definitions of scale-normalized temporal derivatives [17]

$$\partial_\zeta = \tau^{\gamma_\tau/2}\,\partial_t, \qquad (116)$$

where $\gamma_\tau > 0$, in combination with detection of local extrema over temporal scales, enable temporal scale selection.

Provided that the temporal smoothing kernel is given by a one-dimensional Gaussian kernel or the time-causal limit kernel defined by (111), it can be shown that (i) local extrema over temporal scales computed from the internal temporal or spatio-temporal scale-space representation will reflect a characteristic duration of temporal structures that gave result rise to the feature responses and (ii) transform in scale-covariant way such that if a feature detector responds with a local extremum over temporal scales at some position t_0 and some scale τ_0 for a temporal (or spatio-temporal) signal f, then for a rescaled temporal (or spatio-temporal) signal $f'(t') = f(t)$ for $t' = St$, there will be a maximum over temporal scales at position $t'_0 = St_0$ and scale $\tau'_0 = S^2\tau_0$ [17].

This theoretical result holds for any value of $\gamma_\tau > 0$. If $\gamma_\tau = 1$, then the scale-normalized magnitude values at the extrema over temporal scales are additionally equal.

If we in turn normalize the local temporal or spatio-temporal signals f and f' by temporal rescaling transformations around the temporal moments t_0 and t'_0, respectively, by their respective scale parameters τ_0 and τ'_0, then the rescaled frames $f_{ref}(z) = f(t)$ for $z = t/\sqrt{\tau_0}$ and $f'_{ref}(z') = f'(t')$ for $z' = t'/\sqrt{\tau'_0}$, then the rescaled frames will be equal $f'_{ref}(z') = f_{ref}(z)$ for $z' = z$. This means that any image measurement that is expressed in the scale-normalized frames will be invariant under temporal scaling transformations. (In practice, it is not necessary to perform explicit temporal warping between the two frames—it is sufficient to formulate computational operations that can equivalently be related to such scale-invariant reference frames.) Thus, detection of local extrema over temporal scales of scale-normalized temporal derivatives constitutes a general mechanism for temporal scale selection and for obtaining temporal invariance.

In the area of computer vision, this principle has been explored for temporal signals in [17] and spatio-temporal video in [18]. In a biological architecture, corresponding operations could be implemented in wetware based on receptive fields over multiple temporal channels and an operation that selects the maximum value of the responses in combination with a routing mechanism.

3) Spatial affine invariance: Given that we have computed an affine covariant spatial scale-space representation $L(x;\,\Sigma)$ by convolution with a family of affine Gaussian kernels $g(x;\,\Sigma)$ of the form in Equation (21) in the main paper

$$g(x;\,\Sigma) = \frac{1}{2\pi\sqrt{\det\Sigma}}\,e^{-x^T\Sigma^{-1}x/2}. \qquad (117)$$

(for simplicity of notion, we here suppress the complementary scale argument s in this treatment above and instead incorporate it into the covariance matrix by the replacement $s\Sigma \mapsto \Sigma$), let us construct an image representation in a higher layer called *second-moment matrix* or *structure tensor*, obtained by forming the outer product of the gradient vector $\nabla L = (\partial_{x_1}, \partial_{x_2})^T$ by itself $(\nabla L)(\nabla L)^T$ at every point and then integrating the result over image space using another affine Gaussian kernel as weighting function [2], [3]

$$\mu(x;\,\Sigma_{der},\Sigma_{int}) = \int_{u\in\mathbb{R}^2} (\nabla L)(u;\,\Sigma_{der})\,((\nabla L)(u;\,\Sigma_{der}))^T\,g(x-u;\,\Sigma_{int})\,du. \qquad (118)$$

The resulting image representation will depend upon two covariance matrices, a first covariance matrix Σ_{der} for computing the derivatives, and a second covariance matrix Σ_{int} for performing the second-stage integration after the pointwise non-linearity in the outer product.

Consider next an affine transformation of the spatial domain. Assume that we have two images $f(x)$ and $f'(x')$ that are related by an affine transformation $x' = A\,x$ such that $f'(x') = f(x)$. Then, the second-moment matrices over the two affine related spatial domains will be related according to (see [2, Section 15.3.1] [3, Section 5.1] for a proof)

$$\mu'(A\,x;\,A\,\Sigma_{der}\,A^T, A\,\Sigma_{int}\,A^T) = \\ = A^{-T}\,\mu(x;\,\Sigma_{der},\Sigma_{int})\,A^{-1}. \qquad (119)$$

Next, let us assume that we at some image point can determine covariance matrices Σ_{der} and Σ_{int} such that the second-moment matrix is proportional to the inverse of the covariance matrices

$$\mu(x;\,\Sigma_{der},\Sigma_{int}) = c_1\,\Sigma_{der}^{-1} = c_2\,\Sigma_{int}^{-1} \qquad (120)$$

for some constants c_1 and c_2. Then, we obtain a *fixed-point* that can be shown to be *preserved under affine image transformations* (see [3, Section 5.2] for a proof).

Given that we are at such a fixed point in the space of affine parameters, let us determine an affine transformation B from the second-moment matrix u based on the relationship $\mu = c B^T B$, leading to B being proportional to the matrix square root of μ, $B \sim \mu^{1/2}$, and then warping the image f with this transformation, $f''(x'') = f(x)$ for $x'' = Bx$ (see [2, Figure 15.2] or [3, Figure 3] for an illustration). Then, we obtain an essentially affine invariant reference frame, with the second-moment matrix in the transformed frame proportional to the unit matrix $\mu'' = cI$ for some $c > 0$, that is preserved under spatial affine transformations, and with the property that any image measurements performed over this affine invariant reference frame are essentially preserved under affine transformations.

It should be noted, however, that the affine transformation B is not uniquely determined by the fixed-point requirement (118), which only determines two of the four parameters in B, corresponding to amount and direction of perspective foreshortening of a local surface pattern relative to a viewing direction centered on object,

The two remaining degrees of freedom correspond to (i) an overall scaling[11] factor corresponding the viewing distance, which can be determined by scale selection as described in Appendix I1 and (ii) a free rotation[12] angle, corresponding to the selection of a representative direction in the image plane. If the vertical direction is preserved under the perspective transformation, we may not need to perform rotational compensation. Otherwise, orientation normalization can be performed by detecting peaks of gradient directions in a directional histogram and rotationally normalizing the image to these peaks as proposed in [13].

Computationally, affine invariant fixed points that satisfy the property (120) can be reached either by (i) considering a large number of affine Gaussian receptive fields that tesselate the viewsphere (as shown in Figure 8 in the main paper) and signal if the affine fixed point condition is approximately satisfied, or alternatively either (ii) adapting the covariance matrices Σ to the measured second-moment matrices μ from the relationship $\Sigma \sim \mu^{-1}$ complemented by scale normalization in an iterative manner until the amount of update decreases below a threshold, or (iii) iteratively warping the image data with affine transformation matrices determined from $B \sim \mu^{1/2}$ complemented by scale normalization until the second-moment matrix of the resulting composed affine transformed image patch is sufficiently close to being proportional to the unit matrix. In the area of computer vision, the latter two processes are referred to as affine shape adaptation [3]. Typically, only a few iterations is often sufficient to get reasonably close to the affine invariant fixed point.

In the area of computer vision, this affine normalization principle has been used for computing affine invariant image features and image descriptors, typically in the context of performing image matching between different views of a 3-D scene or deriving 3-D shape cues from image data [3], [19], [12], [20], [21], [22].

In a biological architecture, one could consider implementing multiple affine Gaussian receptive fields corresponding to a tesselation on the hemisphere and then having higher level neurons signal how close they are to the fixed-point requirement or some other criterion with similar properties of determining how well the measured local image structure match an internal model of the image data. Then, by selecting the neurons over the parameter space with the best similarity to the fixed-point criterion (or some other criterion with similar properties) and combining this with a routing mechanism, it follows that the visual operations can be invariant to local linearizations of the perspective distortions corresponding to different slant and tilt angles.

4) Galilean invariance: Given that we have computed a Galilean covariant spatio-temporal scale-space representation L by convolution with a family of velocity-adapted kernels of the form (72), let us construct an image representation at a higher layer called *spatio-temporal second-moment matrix* or *spatio-temporal structure tensor* by first forming the outer product $(\nabla L)(\nabla L)^T$ of the spatio-temporal gradient vector $\nabla L = (\partial_{x1} L, \partial_{x_2} L, \partial_t L)$

$$(\nabla L)(p;\, P_{der}) = \nabla \left(\int_{q \in \mathbb{R}^3} f(u)\, T_{der}(p - q;\, P_{der})\, dq \right) \tag{121}$$

by itself at every point $p = (x_1, x_2, t)$ in space-time and then integrating the result over the spatio-temporal domain using another spatio-temporal smoothing kernel of the form (72) as weighting function

$$\mu(p;\, P_{der}, P_{int}) = \int_{q \in \mathbb{R}^3} (\nabla L)(q;\, P_{der})\, ((\nabla L)(q;\, P_{der}))^T\, T_{int}(p-q;\, P_{int})\, dq. \tag{122}$$

The resulting image representation will depend on both the parameters $P_{der} = (v_{der}, s_{der}, \Sigma_{der}, \tau_{der})$

[11] Since the fixed-point condition is determined by the criterion that the second-moment matrix should be proportional to the inverse of the covariance matrix (120), it follows that if we determine an affine transformation B from the relationship $c B^T B = \mu$ as shown in [2, Figure 15.2] or [3, Figure 3], then also any affine transformation $C = c' B$ for some $c' > 0$ also satisfies the fixed-point requirement. Thus, the affine normalization is undetermined up to an arbitrary scaling factor.

[12] To see that the affine normalized frames are related up to an arbitrary rotation, we can proceed as follows: If we transform the pattern $f(x)$ having second-moment matrix μ with the affine transformation $B = \mu^{1/2}$ determined from the condition $B^T B = \mu$, we can first note that also $B = R \mu^{1/2}$ for an arbitrary rotation matrix is a possible solution to the equation $B^T B = \mu$. Correspondingly, if we for the affine related patch $f'(x') = f(x)$ for $x' = Ax$ having second-moment matrix μ' determine an affine transformation B' from the relationship $B'^T B' = \mu'$, we have that $B' = R' \mu'^{1/2}$ is a possible solution for some other arbitrary rotation matrix R'. From the transformation property $\mu' = A^{-T} \mu A^{-1}$ (119), we have that $\mu'^{1/2}$ is a solution to $(A^{-T} \mu^{1/2})^T (\mu^{1/2} A^{-1}) = (\mu'^{1/2})^T (\mu'^{1/2})$, which gives $\mu'^{1/2} = \mu^{1/2} A^{-1}$ implying that $B' = R'(\mu^{1/2} A^{-1}) = R'(R^{-1} B) = R'' B A^{-1}$. If we in turn transform the domain x by this transformation B', we obtain $y' = B'x' = R'' B A^{-1} A x = R'' B x$, while we if we transform the domain x by the transformation B, we obtain $y = Bx$. Thus, $y' = R'' y$ and the the affine normalized frames are equal up a rotation determined by some arbitrary rotation matrix $R'' = R' R^{-1}$. In addition, there is also an arbitrary scaling factor to be determined, as described above.

of the first-stage kernel T_{der} used for computing spatio-temporal derivatives and the parameters $P_{int} = (v_{int}, s_{int}, \Sigma_{int}, \tau_{int})$ of the second-stage kernel T_{int} used for performing the second-stage integration after the pointwise non-linearity in the outer product.

For simplicity, we assume that the spatial covariance matrices in the two layers have similar shape $\Sigma_{int} = \Sigma_{der}$ and that the velocity values over the two domain have similar directions and are related in a similar way as for spatial scale covariance $v_{int}/\sqrt{s_{int}} = v_{der}/\sqrt{s_{der}}$ (see Appendix E) and that the temporal duration is coupled to the spatial extent according to $s_{int}/\tau_{int} = s_{der}/\tau_{der}$.

Consider two Galilean-related spatio-temporal image patterns $f'(p') = f(p)$ that are related by a relative image velocity u such that $p' = G_u\, p$ for a Galilean transformation matrix G_u according to (79). Then, it can be shown that the corresponding spatio-temporal covariance matrices are related according to [1, equation (193)]

$$\mu' = G_u^{-T}\, \mu\, G_u^{-1}. \tag{123}$$

Let us introduce the notion of *Galilean diagonalization*, which corresponds to finding the unique Galilean transformation that transforms the spatio-temporal second-moment matrix to block diagonal form with all mixed purely spatio-temporal components being zero $\mu'_{x_1 t} = \mu'_{x_2 t} = 0$ [23]

$$\mu' = \left(\begin{array}{ccc} \mu'_{x_1 x_1} & \mu'_{x_1 x_2} & 0 \\ \mu'_{x_1 x_2} & \mu'_{x_2 x_2} & 0 \\ 0 & 0 & \mu'_{tt} \end{array} \right). \tag{124}$$

Such a block diagonalization can be obtained if the velocity vector u satisfies

$$\left(\begin{array}{cc} \mu_{x_1 x_1} & \mu_{x_1 x_2} \\ \mu_{x_1 x_2} & \mu_{x_2 x_2} \end{array} \right) \left(\begin{array}{c} u_1 \\ u_2 \end{array} \right) = - \left(\begin{array}{c} \mu_{x_1 t} \\ \mu_{x_2 t} \end{array} \right) \tag{125}$$

with the solution

$$u = -\{\mu_{xx}\}^{-1} \{\mu_{xt}\}, \tag{126}$$

which are structurally similar equations as are used for computing optic flow according to the method by Lukas and Kanade [24]. It can then be shown that the property of Galilean block diagonalization is preserved under Galilean transformations [1, appendix C.4]. Specifically, the velocity vector associated with the Galilean transformation, that brings a second-moment matrix into block diagonal form, is additive under superimposed Galilean transformations. With respect to invariance properties, all spatio-temporal receptive field responses that can be expressed with respect to such a *Galilean invariant spatio-temporal reference frame* will be Galilean invariant.

These ideas have been applied in computer vision for performing spatio-temporal recognition under unknown relative motions between the spatio-temporal events and the observer [25], [26].

Again, it is not necessary to carry out the spatio-temporal normalization in practice to achieve Galilean invariance. In a biological architecture based on a family of spatio-temporal receptive fields that operate over some set of image velocities in parallel, one may consider a routing mechanism that selects receptive field responses by judging the degree of agreement with the criterion of Galilean diagonalization (124) and then giving priority to the responses that are most consistent with this criterion.

References

[1] T. Lindeberg, "Generalized Gaussian scale-space axiomatics comprising linear scale-space, affine scale-space and spatio-temporal scale-space," *Journal of Mathematical Imaging and Vision*, vol. 40, no. 1, pp. 36–81, 2011.

[2] ——, *Scale-Space Theory in Computer Vision*. Springer, 1993.

[3] T. Lindeberg and J. Gårding, "Shape-adapted smoothing in estimation of 3-D depth cues from affine distortions of local 2-D structure," *Image and Vision Computing*, vol. 15, pp. 415–434, 1997.

[4] I. J. Schoenberg, "On Pòlya frequency functions. ii. Variation-diminishing integral operators of the convolution type," *Acta Sci. Math. (Szeged)*, vol. 12, pp. 97–106, 1950.

[5] T. Lindeberg, "Time-causal and time-recursive spatio-temporal receptive fields," *Journal of Mathematical Imaging and Vision*, vol. 55, no. 1, pp. 50–88, 2016.

[6] ——, "Invariance of visual operations at the level of receptive fields," *PLOS ONE*, vol. 8, no. 7, p. e66990, 2013.

[7] ——, "Feature detection with automatic scale selection," *International Journal of Computer Vision*, vol. 30, no. 2, pp. 77–116, 1998.

[8] ——, "Edge detection and ridge detection with automatic scale selection," *International Journal of Computer Vision*, vol. 30, no. 2, pp. 117–154, 1998.

[9] ——, "Scale selection," in *Computer Vision*, K. Ikeuchi, Ed. Springer, 2021, doi:10.1007/978-3-030-03243-2_242-1.

[10] L. Bretzner and T. Lindeberg, "Feature tracking with automatic selection of spatial scales," *Computer Vision and Image Understanding*, vol. 71, no. 3, pp. 385–392, Sep. 1998.

[11] O. Chomat, V. de Verdiere, D. Hall, and J. Crowley, "Local scale selection for Gaussian based description techniques," in *Proc. European Conf. on Computer Vision (ECCV 2000)*, ser. Springer LNCS, vol. 1842, Dublin, Ireland, 2000, pp. I:117–133.

[12] K. Mikolajczyk and C. Schmid, "Scale and affine invariant interest point detectors," *International Journal of Computer Vision*, vol. 60, no. 1, pp. 63–86, 2004.

[13] D. G. Lowe, "Distinctive image features from scale-invariant keypoints," *International Journal of Computer Vision*, vol. 60, no. 2, pp. 91–110, 2004.

[14] H. Bay, A. Ess, T. Tuytelaars, and L. van Gool, "Speeded up robust features (SURF)," *Computer Vision and Image Understanding*, vol. 110, no. 3, pp. 346–359, 2008.

[15] T. Tuytelaars and K. Mikolajczyk, *A Survey on Local Invariant Features*, ser. Foundations and Trends in Computer Graphics and Vision. Now Publishers, 2008, vol. 3(3).

[16] T. Lindeberg, "Image matching using generalized scale-space interest points," *Journal of Mathematical Imaging and Vision*, vol. 52, no. 1, pp. 3–36, 2015.

[17] ——, "Temporal scale selection in time-causal scale space," *Journal of Mathematical Imaging and Vision*, vol. 58, no. 1, pp. 57–101, 2017.

[18] ——, "Spatio-temporal scale selection in video data," *Journal of Mathematical Imaging and Vision*, vol. 60, no. 4, pp. 525–562, 2018.

[19] A. Baumberg, "Reliable feature matching across widely separated views," in *Proc. Computer Vision and Pattern Recognition (CVPR'00)*, Hilton Head, SC, 2000, pp. I:1774–1781.

[20] T. Tuytelaars and L. van Gool, "Matching widely separated views based on affine invariant regions," *International Journal of Computer Vision*, vol. 59, no. 1, pp. 61–85, 2004.

[21] S. Lazebnik, C. Schmid, and J. Ponce, "A sparse texture representation using local affine regions," *IEEE Trans. Pattern Analysis and Machine Intell.*, vol. 27, no. 8, pp. 1265–1278, 2005.

[22] F. Rothganger, S. Lazebnik, C. Schmid, and J. Ponce, "3D object modeling and recognition using local affine-invariant image descriptors and multi-view spatial constraints," *International Journal of Computer Vision*, vol. 66, no. 3, pp. 231–259, 2006.

[23] T. Lindeberg, A. Akbarzadeh, and I. Laptev, "Galilean-corrected spatio-temporal interest operators," in *Int. Conf. on Pattern Recognition*, Cambridge, 2004, pp. I:57–62.

[24] B. D. Lukas and T. Kanade, "An iterative image registration technique with an application to stereo vision," in *Image Understanding Workshop*, 1981.

[25] I. Laptev and T. Lindeberg, "Velocity-adapted spatio-temporal receptive fields for direct recognition of activities," *Image and Vision Computing*, vol. 22, no. 2, pp. 105–116, 2004.

[26] I. Laptev, B. Caputo, C. Schuldt, and T. Lindeberg, "Local velocity-adapted motion events for spatio-temporal recognition," *Computer Vision and Image Understanding*, vol. 108, pp. 207–229, 2007.
